# A Genomic Survey of *SCPP* Family Genes in Fishes Provides Novel Insights into the Evolution of Fish Scales

**DOI:** 10.3390/ijms18112432

**Published:** 2017-11-16

**Authors:** Yunyun Lv, Kazuhiko Kawasaki, Jia Li, Yanping Li, Chao Bian, Yu Huang, Xinxin You, Qiong Shi

**Affiliations:** 1 BGI Education Center, University of Chinese Academy of Sciences, Shenzhen 518083, China; lvyunyun@genomics.cn (Y.L.); huangyu@genomics.cn (Y.H.); youxinxin@genomics.cn (X.Y.); 2 Shenzhen Key Lab of Marine Genomics, Guangdong Provincial Key Lab of Molecular Breeding in Marine Economic Animals, BGI Academy of Marine Sciences, BGI Marine, BGI, Shenzhen 518083, China; lijia1@genomics.cn (J.L.); liyanping@genomics.cn (Y.L.); bianchao@genomics.cn (C.B.); 3 Department of Anthropology, Penn State University, University Park, PA 16802, USA; kuk2@psu.edu; 4 Laboratory of Aquatic Genomics, College of Life Sciences and Oceanography, Shenzhen University, Shenzhen 518060, China

**Keywords:** *SCPP* gene, evolution, scale, cavefish, mutation

## Abstract

The family of secretory calcium-binding phosphoproteins (SCPPs) have been considered vital to skeletal tissue mineralization. However, most previous *SCPP* studies focused on phylogenetically distant animals but not on those closely related species. Here we provide novel insights into the coevolution of *SCPP* genes and fish scales in 10 species from *Otophysi*. According to their scale phenotypes, these fishes can be divided into three groups, i.e., scaled, sparsely scaled, and scaleless. We identified homologous *SCPP* genes in the genomes of these species and revealed an absence of some *SCPP* members in some genomes, suggesting an uneven evolutionary history of *SCPP* genes in fishes. In addition, most of these *SCPP* genes, with the exception of *SPP1*, individually form one or two gene cluster(s) on each corresponding genome. Furthermore, we constructed phylogenetic trees using maximum likelihood method to estimate their evolution. The phylogenetic topology mostly supports two subclasses in some species, such as *Cyprinus carpio*, *Sinocyclocheilus anshuiensis*, *S. grahamin*, and *S. rhinocerous*, but not in the other examined fishes. By comparing the gene structures of recently reported candidate genes, *SCPP1* and *SCPP5*, for determining scale phenotypes, we found that the hypothesis is suitable for *Astyanax mexicanus*, but denied by *S. anshuiensis*, even though they are both sparsely scaled for cave adaptation. Thus, we conclude that, although different fish species display similar scale phenotypes, the underlying genetic changes however might be diverse. In summary, this paper accelerates the recognition of the *SCPP* family in teleosts for potential scale evolution.

## 1. Introduction

Phenotypic variations underlying the genetic differences between species are cryptic and attractive for biologists. The appearance of new gene(s) leading to innovative feature(s) can provide organisms with capacities to adapt to natural challenges [1]. The secretory calcium-binding phosphoprotein (SCPP) family have attracted special attention, mainly due to their crucial functions in the mineralization of bone, dentin, enamel, and enameloid, which are beneficial to vertebrates’ protection, predation, and locomotion [2,3,4]. These genes arose from gene duplication and originated from a common ancestor, *SPARCL1* (the secreted protein, acidic, cysteine-rich like 1). According to their chemical constitutions, the SCPPs fall into two subclasses: acidic SCPPs containing >25% of Glu (E), Asp (A), and phospho-Ser (pS) residues and Pro/Gln (P/Q)-rich SCPPs consisting of >20% of Pro and Gln [5]. Acidic SCPPs are reported to participate in bone and dentin mineralization, whereas P/Q-rich SCPPs are involved in enamel mineralization [4].

Studies on fish genomes have paid attention to differences in the repertoire of *SCPP* genes that probably correlate with phenotypic transitions of mineralized tissues, and interesting and profound biological implications about the *SCPP* family have been reported. In elephant shark, a cartilaginous fish that diverged early from the bony fish lineage, only two *SCPP*-related ancestral genes (i.e., *SPARC* and *SPARCL1*) were identified in its genome, suggesting earlier origins compared to *SCPP* genes found in bony fishes [6]. The absence of *SCPP* genes in the elephant shark genome may account for the cartilaginous features of skeleton in chondrichthyes, which are in contrast to relatively higher degrees of mineralized skeleton in bony fish, including teleosts. Moreover, targeted mutagenesis of *SPP1,* an acidic protein in SCPPs, in zebrafish led to a reduction in bone formation [6]. This result suggests that the loss of *SCPP* genes would affect the process of skeletal mineralization, and corroborates the essential function of *SPP1* in bone formation. In tiger tail seahorse, two acidic *SCPP* genes (*SCPP1* and *SPP1*) were retained but P/Q-rich genes, associated with dentine and enameloid mineralization, were entirely missing in the genome, suggesting that P/Q-rich *SCPP* genes are presumably associated with their tooth loss [7].

**Figure 1:**
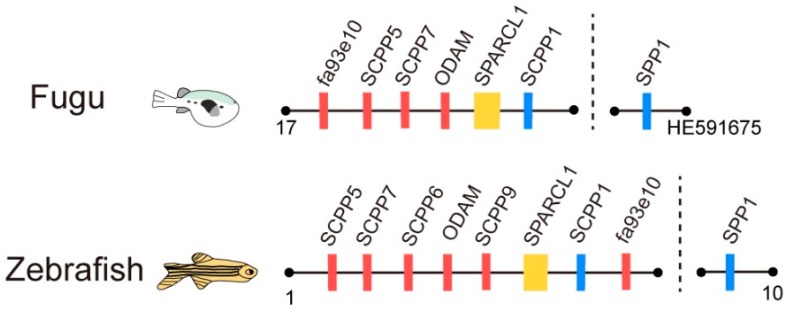
The *SPARCL1*-*SCPP* cluster in fugu and zebrafish (modified from [10,11]).

In vertebrates, two *SCPP* genes, *SPP1* and *ODAM*, and their common ancestor, *SPARACL1*, are conserved in jawed vertebrates with a significant similarity in encoded amino acid sequences between tetrapods and teleosts [8,9]. However, other members of the *SCPP* family are likely to be more specific between lineages, indicating that they arose with lineage specific duplications and deletions, which possibly results in the specialization of certain *SCPP* family genes in vertebrates. Although the specialization of *SCPP* family members existed between tetrapods and teleosts, the feature whereby the *SCPP* family forms a gene cluster is shared by genomes. In fishes such as fugu [10] and zebrafish [11], *SPARCL1* and other *SCPP* genes (except for *SPP1*) constitute the *SPARCL1-SCPP* gene cluster (Figure 1). This cluster was also confirmed in coelacanth [9], spotted gar [8], and sunfish [12]. However, the knowledge of *SPARCL1-SCPP* gene cluster was limited to a few fishes as mentioned above, which hampers a deeper understanding of its evolution in teleosts.

A recent report showed the upregulation of *SCPP* genes during scale development in common carp, providing reliable evidence that *SCPP* genes are involved in the regulation of scale development in fishes [13]. The study further compared the existence of *SCPP* genes in scaled and scaleless fishes, and observed loss of *SCPP1* and/or *SCPP5* in scaleless fish, but the presence of them were exhibited in scaled fishes. Therefore, these two genes were regarded as candidates for determining scale phenotypes. Besides the strict scaled or scaleless phenotype in fishes, there is another pattern of phenotype called “sparsely scaled” feature, and it presents in fishes such as Mexican tetra (*Astyanax mexicanus*) [14] and Chinese golden-line fishes (*Sinocyclocheilus rhinocerous* and *S. anshuiensis*) [15]. *A. mexicanus* and *S. anshuiensis* are cave-restricted fishes, while *S. rhinocerous* is semi-cave-dwelling. These species exhibit sparse scales in their surface skin, whereas their relatives living in surface rivers present rich scales. Therefore, the functions of *SCPP* genes in determining scale phenotypes need to be further verified in sparsely scaled fishes. However, no such studies are available currently.

This present study focuses on the *SCPP* repertoire in 10 fishes, including *A. mexicanus* (abbreviated as AM), *Ctenopharyngodon idellus* (CI; grass carp), *Cyprinus carpio* (CC; common carp), *Ictalurus punctatus* (IP; Channel catfish), *Leuciscus waleckii* (LW; Amur ide), *Pimephales promelas* (PP; fathead minnow), *Pygocentrus nattereri* (PN; red-bellied piranha), *Sinocyclocheilus anshuiensis* (SA), *Sinocyclocheilus grahamin* (SG), and *Sinocyclocheilus rhinocerous* (SR). All species belong to the Series *Otophysi* and are classified as Cypriniformes, Characiformes, or Siluriformes. In terms of morphology, they present three scale phenotypes and can be categorized as scaled (including CI, CC, LW, PP, PN, and SG), scaleless (such as IP), or sparsely scaled (such as AM, SR and SA) fishes. The main purpose of our present research is to enrich the evolutionary studies of *SPARCL1-SCPP* cluster in certain fish lineages. We focused on sparsely scaled fishes to test the roles of *SCPP1* and *SCPP5* as candidate genes for scale phenotype determination. In this paper, we concentrate on *SPARCL1* and *SCPP* genes, and attempt to answer three core questions: (1) Does the reported *SPARCL1-SCPP* cluster display a similar arrangement in the examined fishes? (2) How do the *SPARCL1* and SCPP genes evolve among species? (3) Does the hypothesis of *SCPP1* and *SCPP5* serving as candidate genes for determining scale phenotypes work for the sparsely scaled species?

## 2. Results

### 2.1. Collection of SPARCL1 and SCPP Genes and Transcriptome Confirmation

**Figure 2:**
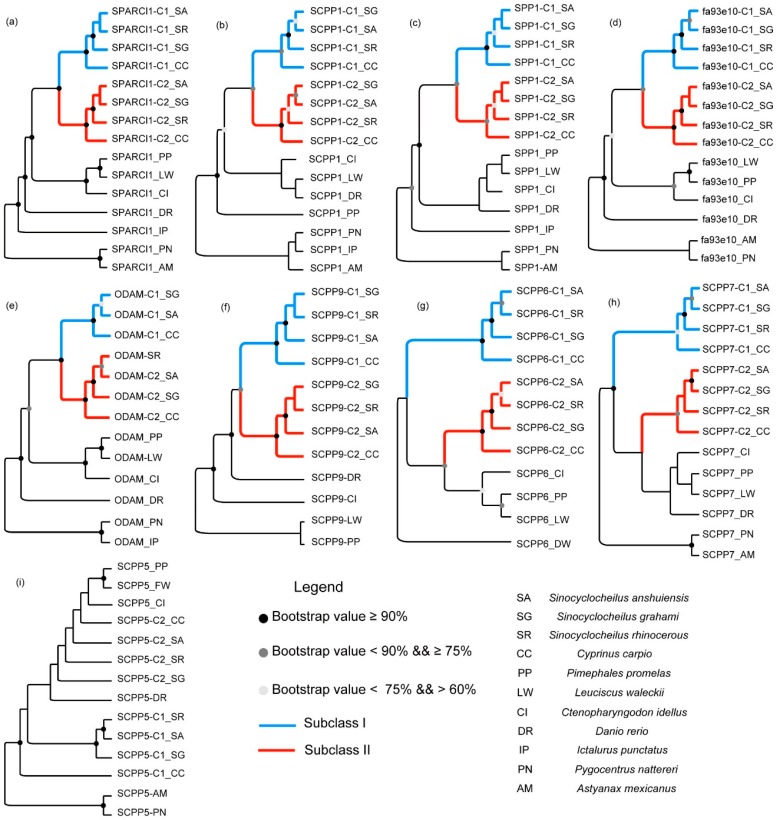
Phylogeny of *SPARCL1* and eight *SCPP* family genes among 11 fish species. The sequences from zebrafish (*Danio rerio*; DR) were downloaded from NCBI (find detailed accession numbers in Table S1) for subsequent homology searching, and other sequences were extracted from corresponding genomes. (**a**–**i**) The phylogenic topology of *SPARCL1*, *SCPP1*, *SPP1*, *fa93e10*, *ODAM*, *SCPP9*, *SCPP6*, *SCPP7*, and *SCPP5*, respectively. The blue and red shaded branches mark Subclasses I and II, respectively, among the two gene copies within CC, SA, SR, and SG. The phylogenetic analysis using maximum likelihood (ML) methods was performed, replicated 1000 times, to evaluate their branch supports, which are displayed as circles in the nodes when higher than 60%.

We obtained a total of 115 nucleotide sequences for 14 *SPARCL1* genes (Figure S1) and 101 *SCPP* family genes (including *SPP1*, *SCPP1*, *ODAM*, *fa93e10*, *SCPP5*, *SCPP6*, *SCPP7*, and *SCPP9*; Figures S2–S9). All these sequences were derived from 10 fish species, namely, AM, CI, CC, IP, LW, PP, PN, SA, SG, and SR, using the sequences from zebrafish (DR) as the queries (Figure 2). Multiple alignment of the nucleotide sequences for each gene displays high similarities among species (see more details in Figures S1–S9).

In *Sinocyclocheilus* species (SA, SG, SR) and CC, *SPARCL1* and *SCPP* genes were found to be doubled, with an exception of only one copy of *ODAM* in SR. In contrast, only a single copy of *SPARCL1* and *SCPP* genes was identified in other fishes (PP, LW, CI, IP, PN, and AM) as the same as spotted gar [8] and zebrafish [11]. Thus, double copies of *SPARCL1* and *SCPP* genes in SA, SG, SR, and CC may imply that these genes had ever undergone a gene-duplication event. Our predicted *SPARCL1* and *SCPP* genes in SG, SR, and SA were mostly supported by transcriptome assemblies [15] (see more details in Section 4.1), but with the exception for the *SCPP5* gene.

Despite the success of collecting all genes in Cypriniformes, we failed to identify some of the *SCPP* genes from the genomes of species in Siluriformes and Characiformes, such as IP, AM, and PN. In IP, only *SPARCL1*, *SCPP1*, *SPP1*, and *ODAM* were identified, whereas more genes were identified in AM and PN, although *SCPP6* and *SCPP9* were still missing. It seems that the distribution of *SCPP* genes in the genomes of these teleost species is uneven, and certain *SCPP* genes may be evolved with lineage-specific activities.

### 2.2. Phylogenetic Topologies of SPARCL1 and SCPP Genes

The topology of evolutionary trees deduced from the alignments of *SPARCL1*, *SCPP1*, *SPP1*, *fa93e10*, *ODAM*, and *SCPP9* commonly exhibits two major groups (Subclasses I and II in Figure 2) among SG, SA, SR, and CC, but with the exceptions of *SCPP6*, *SCPP7,* and *SCPP5*, suggesting that the evolution of these genes was not coincided.

In the phylogeny of *SPARCL1*, *SCPP1*, *SPP1*, *fa93e10*, *ODAM*, and *SCPP9*, Subclass I and Subclass II form sister groups (Figure 2a–f), indicating a closer relationship of these genes than the others. For *SCPP6* and *SCPP7*, although two groups among SG, SA, SR, and CC were still presented, they did not form sister groups yet (Figure 2g,h). The different topologies of the Subclasses I and II may result from a higher nucleotide substitution rate of *SCPP6* and *SCPP7* in one subclass. In addition, the phylogenetic topology of *SCPP5* (Figure 2i) is completely different from other *SCPP* genes, suggesting a more complex evolutionary history compared with other *SCPP* genes.

### 2.3. The Putative SPARCL1-SCPP Cluster and Pseudogenes

**Figure 3:**
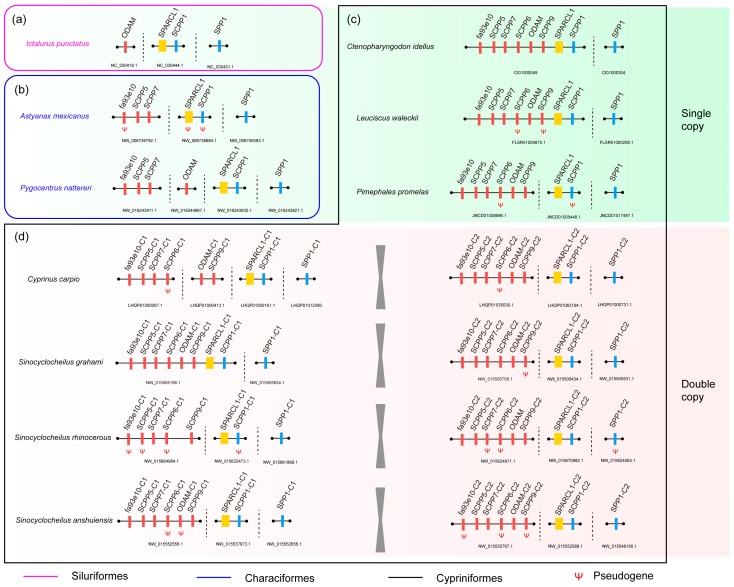
The genomic arrangement of *SPARCL1* and *SCPP* genes in the 10 examined fish genomes. (**a**,**b**,**c**,**d**) Siluriformes, Characiformes, and Cypriniformes fishes, respectively. The yellow, red, and blue colors represent the ancestral *SPARCL1* gene, acidic SCPP-encoding genes, and P/Q-rich SCPP-encoding genes, respectively. “ψ” stands for possible pseudogenes with missing exons, codon frameshifts, or premature stop codons.

By mapping the *SPARCL1* and *SCPP* sequences onto the 10 examined fish genomes, we observed that most of them are localized into a common region with the formation of a single or multiple gene cluster(s) (Figure 3). The single cluster includes *fa93e10*, *SCPP5*, *SCPP7*, *SCPP6*, *ODAM*, *SCPP9*, *SPARCL1*, and *SCPP1* in CI and LW (Figure 3c), similar to the localization in the reported zebrafish genome [11]. While in IP, AM, PN, PP, CC, SG, SR, and SA, the *SCPP* genes were separated into two clusters, including a relatively long one and another short one. The long one includes *fa93e10*, *SCPP5*, *SCPP7*, *SCPP6*, *SCPP9*, and *ODAM*, and the short one consists of *SPARCL1* and *SCPP1* (Figure 3). These different genomic arrangements indicate that the *SPARCL1*-*SCPP* cluster within these fish species may be various in evolution.

Interestingly, *SPP1* is always separately distributed in the 10 examined genomes, suggesting its separation from the main gene cluster in the early evolution, which has been previously reported in fugu [10] and zebrafish [11].

By comparing the predicted gene structures of these collected genes, we considered those predicted genes with missing exons, codon frameshifts, or premature stop codons as possible pseudogenes (Figure 3b–d). Interestingly, we observed more possible pseudogenes in these sparsely scaled species (AM, SR, and SA). In AM, half of the *SCPP*s *(SPARCL1*, *SCPP1*, and *fa93e10*) were identified as possible pseudogenes (Figure 3b). Among the three *Sinocyclocheilus* fishes, only one possible pseudogene (*SCPP9-*C2) was identified in the surface-dwelling SG, but there are seven and five possible pseudogenes in the semi-cave-dwelling SR and cave-restricted SA, respectively (Figure 3d).

### 2.4. SCPP1 and SCPP5: Gene Structure Comparison between Cavefishes and Other Fishes

**Figure 4:**
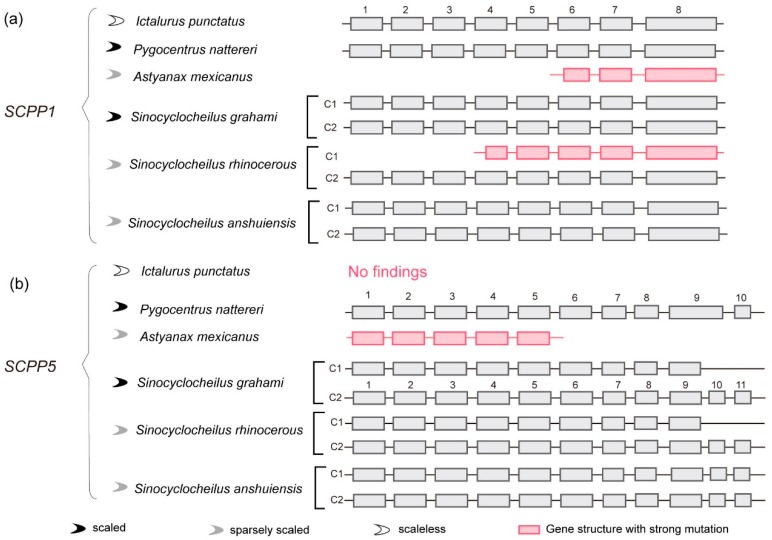
Comparison of candidate genes for determining scale phenotype among scaled, sparsely scaled, and scaleless fishes. (**a**,**b**) Structural alignments of *SCPP1* and *SCPP5* genes, respectively, in six representative fishes, namely, scaled PN and SG, sparsely scaled AM, SR, and SA, and scaleless IP.

To test the hypothesis of *SCPP1* and *SCPP5* as candidate genes for determining scale phenotypes [13], we chose and compared fishes with scaled, sparsely scaled, and scaleless phenotypes (Figure 4). Interestingly, we found that the first five and three exons in *SCPP1* of sparsely scaled AM and SR, respectively, were missing (Figure 4a), and the last five exons in *SCPP1* of sparsely scaled AM were as well (Figure 4b). These structural changes suggest their pseudogene status. We did observe that *SCPP1* and *SCPP5* were quite normal in another sparsely scaled species SA, but no *SCPP5* was identified in the scaleless IP.

In the three *Sinocyclocheilus* fishes, an extra exon appeared (Figure 4b). Based on the pairwise alignment of *SCPP1* between them and zebrafish, we evaluated that this additional exon was generated by a crack of the original Exon 9. We temporarily regard these *SCPP5s* as normal genes, although we cannot determine whether this crack causes pseudogenization.

## 3. Discussion

### 3.1. Comparison of the SPARCL1-SCPP Cluster in Teleosts

Since the report of *SPARCL1*-*SCPP* cluster in fugu [10], similar genomic arrangement was confirmed in zebrafish [11], coelacanth [9], spotted gar [8], and sunfish [12]. In our present work, this pattern was further corroborated (Figure 3c) in grass carp (CI) and Amur ide (LW). However, we observed a division of the putative *SPARCL1*-*SCPP* cluster into two segments in other examined fishes, with one portion consisting of *SPARCL1* and *SCPP1* and the other composed of *fa93e10*, *SCPP5*, *SCPP7*, *SCPP6*, *ODAM*, and *SCPP9* (see more details in Figure 3). The crack of *SPARCL1*-*SCPP* cluster indicates chromosomal rearrangement in these fishes.

Furthermore, we compared a copy number of *SPARCL1* and *SCPP* genes in fishes including Siluriformes, Characiformes and Cypriniformes. Siluriformes, such as IP, own the least number of such genes with only *SPARCL1*, *ODAM*, *SCPP1*, and *SPP1* were identified (Figure 3a). Characiformes (such as AM and PN) have a medium number of *SCPP*s between Siluriformes and Cypriniformes (Figure 3b). The various amount of *SCPP* gene numbers among different groups suggests that some *SCPP* genes are lineage-specific and the evolutionary history of *SCPP*s in certain families of teleosts is uneven. Interestingly, among the eight *SCPP* genes, *SCPP6* only existed in Cypriniformes, but disappeared in Siluriformes and Characiformes, implying a special role of *SCPP6* in Cypriniformes. The loss of *SCPP6* was also reported previously in fugu [10], sunfish [12], coelacanth [9], and spotted gar [8]. Taken together, it seems that *SCPP6* is very unique with only existence in Cypriniformes. Thus, in addition to the previous report of the independent evolution of *SCPP* genes between teleosts and tetrapods [2], we also demonstrate that the evolution of the *SPARCL1*-*SCPP* cluster among lineages in teleosts are unparallel, with an independent history of certain *SCPP* genes.

### 3.2. Evolution of SPARCL1 and SCPP Genes during Whole Genome Duplication

In contrast to the richness of *SCPP* genes in teleosts, the *SCPP* family was absent in cartilaginous fishes (such as little skate, the small-spotted catshark, and elephant shark) and jawless vertebrates (such as sea lamprey) [6]. This indicates that the primary vertebrates had unevolved the *SCPP* family until the appearance of teleosts. Thus, *SCPP* genes existed in teleosts were thought to be evolved after the actinopterygian–sarcopterygian divergence [11]. In vertebrates, as we know, two rounds of whole-genome duplication (WGD) occurred in the common ancestor [16,17]. More specifically, the two rounds of WGD happened before the agnatha–gnatostoma and chrondrichthyes–osteichthyes split, respectively. The third round of WGD was specific to teleosts (TSGD) that occurred after the actinopterygian–sarcopterygian split [18,19]. Thereby, *SCPP* genes in teleosts are probably originated from TSGD and duplicated from the *SPARCL1* gene. Subsequently, *SCPP* genes evolved along with the course of speciation and certain *SCPP* genes arose in lineage-specific ways.

Besides the above-mentioned three rounds of WGD, some fish lineages such as Acipenseridae, Catostomidae, Cobitidae, Cyprininae, and Salmonidae underwent a fourth round of WGD [20]. The genomic studies of common carp and *Sinocyclocheilus* species [15,20] indicate their tetraploid nature in Cyprininae. Thus, the double copies of *SPARCL1* and *SCPP* genes in CC, SG, SR, and SA (Figure 3) were presented with a high possibility of yielding in the fourth Cyprininae-specific WGD. WGD was thought to provide raw genetic materials for new gene appearance, allowing organisms to acquire new traits to survive in the natural challenges [1]. TSGD-originated *SCPP* genes have proven to be crucial for tissue mineralization, such as the mineralizing process of tooth, bone, and scale formation [6,10,11,13]. These tissues help teleosts with predation, locomotion, digestion, and protection, which are the basic skills for survival. In contrast to the initially arisen *SCPP* genes, the newly yielded *SCPP* copies in the fourth WGD of Cypriniformes were reported for the first time in our present study. The exact functions of these new *SCPP* copies are still unknown, although *SCPP7* and *fa93e10* were reported to have much higher transcription during scale regeneration in common carp (CC) [13].

In general, the fate of WGD duplications can be either pseudogenization, subfunctionalization, or neofunctionalization. It has been reported that rapid gene loss did occur after the TSGD, and most genes were doubled in this event and were subsequently lost quickly in the initial 60 Ma after TSGD [21]. However, the fate of duplicated genes in the fourth round of WGD in Cyprininae has been unknown yet. From the perspective of *SPARCL1* and *SCPP* genes, we found basically two copies in CC, SG, SR, and SA, despite the possibility that one copy of *ODMA* in SR was missing. This suggests the fate of duplicated *SPARCL1* and *SCPP* genes in the fourth WGD may differ from the fast lost gene copies yielded in TSGD.

However, the structural changes displayed in some duplicated *SCPP* genes of CC, SG, SR, and SA (Figure 3d and Figure 4) suggest their possible transformation into pseudogenes. Thus, some duplicated *SCPP* genes faced fast functional loss after the fourth WGD. In contrast to the proportion of gene copies into pseudogenes, more *SCPP* gene copies were found as normal as those in zebrafish with similar gene structures (Figure 4). Therefore, this part of duplicated *SCPP* genes in the fourth WGD might be retained for neofunctionalization or subfunctionalization. Recently reported Atlantic salmon genome provides novel insights into gene fate after the fourth WGD in Salmonidae [22]. The fate of duplicates of salmonids in the fourth WGD likely prefers neofunctionalization to subfunctionalization, which provide salmonids with a wide range of ecological adaptions. According to this pattern, the newly predicated *SCPP* duplicates in Cyprininae may also generate outcomes of functional divergence and benefit their living in evolutionary history, but this needs further experimental verification.

### 3.3. Comparison of SCPP1 and SCPP5 in Scaled, Sparsely Scaled and Scaleless Fishes

Cavefishes are restricted to subterranean environments. Besides the blind eyes and albinism that are obviously different from the surface-dwelling counterparts, cavefishes also show a great decrease in the number of scales [14,15]. Previously, *SCPP1* and/or *SCPP5* have been reported to be candidate genes for determining scale phenotypes [13]. Our present study added two more cave-restricted fishes (AM and SA) and one semi-cave-dwelling SR to compare the gene changes of *SCPP1* and *SCPP5*. Our results revealed structural changes in both *SCPP1* and *SCPP5* in AM but not in another cavefish SA; some exons in *SCPP1* are missed in the semi-cave-dwelling SR, but not in the cave-restricted SA (Figure 4). These data suggest that the gene changes in AM and SA are not uniform, and even in phylogenetically closed species, SR and SA are also uneven, even though their scale phenotypes are similar.

It seems that the hypothesis of *SCPP1* and *SCPP5* regarded as candidate genes for determining scale phenotype is suitable for AM, but denied by SA. Consequently, we estimated that, in addition to *SCPP1* and *SCPP5*, more genes are necessary for involvement in the process of scale development. Although both AM and SA are sparsely scaled, the underlying genetic mechanisms leading to this phenotype could be diverse.

Different changes between AM and SA were also revealed in melanogenesis-related gene *Oca2*. The albinism of AM might be generated because some exon regions of *Oca2* are missed, resulting in the termination of upstream steps of the melanin synthesis pathway [23]. However, such changes were denied by albinotic SA [15]. Thus, given that the functions of *SCPP1*, *SCPP5*, and *Oca2* are related to scales and albinism in AM and SA, we propose that, although fishes from different lineages present convergent evolution, molecular changes of similar phenotypes can be completely different.

## 4. Materials and Methods

### 4.1. Gene Collection and Transcriptome Confirmation

Firstly, the protein sequences (Table S1) of *SPARCL1*, *SPP1*, *SCPP1*, *ODAM*, *fa93e10*, *SCPP5*, *SCPP6*, *SCPP7,* and *SCPP9* identified by Kawasaki (2009) were downloaded from the National Center for Biotechnology Information (NCBI). Secondly, whole genomes of nine fishes (AM, CC, IP, LW, PP, PN, SA, SG, and SR) were downloaded from NCBI, and the genome of grass carp (CI) retrieved from the official National Center for Gene Research website (http://www.ncgr.ac.cn/grasscarp/), to construct a local database (Table S2). Nucleotide sequences of *SPARCL1*, *SPP1*, *SCPP1*, *ODAM*, *fa93e10*, *SCPP5*, *SCPP6*, *SCPP7*, and *SCPP9* were extracted from these genomes using BLAST (version 2.2.28 [24]) and Exonerate (version 2.2.0 [25]). We also provided further confirmation of these extracted *SPARCL1* and *SCPP* genes in skin transcriptome data among SG, SR, and SA. Related transcriptome assemblies were generated in our previous paper [15].

### 4.2. Sequence Alignment and Phylogenetic Reconstruction

The collected nucleotide sequence of *SPARCL1*, *SPP1*, *SCPP1*, *ODAM*, *fa93e10*, *SCPP5*, *SCPP6*, *SCPP7*, and *SCPP9* were processed for phylogenetic analyses. Multiple codon-based alignments of the collected sequences were initially performed using MEGA (version 7.0 [26]) with the Muscle module. Each alignment of genes was subsequently adjusted manually. The final aligned nucleotide sequences were employed to predict their best nucleotide substitution model under the Akaike Information Criterion (AIC) [27], which was implemented in Jmodeltest (version 2.0 [28]). The parameters within the best nucleotide substitution models (GTR+G for *SPARCL1*, *SPP1*, *SCPP1*, *ODAM*, *fa93e10*, *SCPP6,* and *SCPP7*, HKY85+G for *SCPP5*, and GTR+I for *SCPP9*) were applied using PhyML (version 3.1 [29,30]) to construct phylogenetic topologies with the maximum likelihood (ML) method and 1000 replicates for the evaluation of their branch supports.

### 4.3. Genomic Location and Pseudogene Identification

By mapping the collected sequences onto their corresponding genomes, the genomic location of *SPARCL1* and *SCPP* genes were determined, which was implemented in TBtools (https://github.com /CJ-Chen/TBtools). Pairwise alignment of *SPARCL1*, *SPP1*, *SCPP1*, *ODAM*, *fa93e10*, *SCPP5*, *SCPP6*, *SCPP7*, and *SCPP9* between extracted species and zebrafish was also performed in Exonerate software (Figures S1–S9). Missing exon regions, codon frameshifts, or premature stop codon(s) within each gene were identified for the consideration of a possible pseudogene.

## 5. Conclusions

In this paper, we investigated and compared the *SCPP* repertoire from 10 fishes of *Otophysi*. We observed that the diversity of *SCPP* members among various fish lineages was uneven, and certain *SCPP* genes evolved specifically by lineages. After comparing the *SCPP* gene copies, we estimated that the fourth WGD in Cyprininae with a high possibility caused the duplication of *SPARCL1* and *SCPP* genes. However, some duplicated *SCPP* genes changed into pseudogenes, whereas others were retained with structural normality. The previously reported hypothesis considering *SCPP1* and/or *SCPP5* as candidates for scale phenotype determination is suitable for AM, but denied by SA, even though they were both sparsely scaled cavefishes. Through these analyses and comparisons, we provide new insights into teleost *SCPP* family genes for potential scale evolution.

## Supplementary Materials

Supplementary materials can be found at www.mdpi.com/1422-0067/18/11/2432/s1.

## Abbreviations

AM	*Astyanax mexicanus*
CC	*Cyprinus carpio*
CI	*Ctenopharyngodon idellus*
IP	*Ictalurus punctatus*
LW	*Leuciscus waleckii*
*Oca2*	Gene encoding oculocutaneous albinism II (melanocyte-specific transporter protein)
*ODAM*	Gene encoding odontogenic ameloblast-associated protein (Ameloblast-associated odontogenic protein)
PP	*Pimephales promelas*
PN	*Pygocentrus nattereri*
SA	*Sinocyclocheilus anshuiensis*
SG	*Sinocyclocheilus grahamin*
SR	*Sinocyclocheilus rhinocerous*
*SCPP1*	secretory calcium-binding phosphoprotein 1
*SCPP5*	secretory calcium-binding phosphoprotein 5
*SCPP6*	secretory calcium-binding phosphoprotein 6
*SCPP7*	secretory calcium-binding phosphoprotein 7
*SCPP9*	secretory calcium-binding phosphoprotein 9
*SPARC*	secreted protein acidic and rich in cysteine
*SPARCL1*	Gene encoding *SPARC*-like protein 1
*SPP1*	Gene encoding secreted phosphoprotein 1
TSGD	teleosts-specific genome duplication
WGD	whole genome duplication
