# Supplementary material for: A Genomic Survey of SCPP Family Genes in Fishes Provides Novel Insights into the Evolution of Fish Scales
Source: Int J Mol Sci. 2017 Nov 16;18(11):2432. doi: 10.3390/ijms18112432 (PMC5713400; doi:10.3390/ijms18112432)
Supplement: Supplementary file 1 [file Figure S5.SCPP5.cds.pdf › ijms-236743-supplementary/Figure S5.SCPP5.cds.pdf]

Sequence	1–80	
SCPP5_AM	ATGAAATCCCTCGTTTTGTTGGCATGTGTCATTGGAGTGGCCCTCTGCGCTCCGATATCACCATTCTTCAACTACTTGCC	80
SCPP5_PN	ATGTTGGGCTCCATTCTCTGCCTTTCTTTCGTCACAATGGTGTCTGCTGCACCGATGTCACCATTCTTCAACTATTTGCC	80
SCPP5_CI	ATGTGGACATCTATCCTGTGTCTTCTTCTGGCTGGTGCAGTATCCTCAGCACCACTTTCACCGTTTTTCAATTATCTCCC	80
SCPP5_DR	ATGTGGACGTCTCTCCTGTGTCTTCTGCTGGCCGGTGCAGTGTCTGCCGCTCCACTTTCACCGTTTTTCAATTATCTCCC	80
SCPP5_LW	ATGTGGACATCTTTCCTGTGTCTT...CTTGCGGGTGCAGTATCCGCAGCACCACTTTCACCGTTTTTCAATTATCTCCC	77
SCPP5_PP	ATGTGGACATCTATCCTGTGTCTTCTTCTGGCGGGTGCAGTATCCGCAGCACCACTTTCACCGTTTTTCAATTATCTCCC	80
SCPP5-C1_CC	ATGTGGACATCTCTCCTGTGTCTTCTTCTGGCTGGTGCAGTATCTGCAGCACCACTTTCACCATTTTTCAATTATCTCCC	80
SCPP5-C2_CC	ATGTGGACATCTCTCCTGTGTCTTCTTCTGGCTGGTGCAGTATCCGCAGCACCACTTTCACCATTTTTCAATTATCTCCC	80
SCPP5-C1_SA	ATGTGGACATCTCTCCTGTGTCTTCTTCTGGCTGGTGCAGTATCTGCAGCACCACTTTCACCGTTTTTCAATTATCTCCC	80
SCPP5-C2_SA	ATGTGGACATCTCTCCTGTGTCTTCTTCTGGCTGGTGCAGTATCTGCAGCACCACTTTCACCATTTTTCAATTATCTCCC	80
SCPP5-C1_SG	ATGTGGACATCTCTCCTGTGTCTTCTTCTGGCTGGTGCAGTATCTGCAGCACCACTTTCACCGTTTTTCAATTATCTCCC	80
SCPP5-C2_SG	ATGTGGACATCTCTCCTGTGTCTTCTTCTGGCTGGTGCAGTATCCGCAGCACCACTTTCACCATTTTTCAATTATCTCCC	80
SCPP5-C1_SR	ATGTGGACATCTCTCCTGTGTCTTCTTCTGGCTGGTGCAGTATCTGCAGCACCACTTTCACCGTTTTTCAATTATCTCCC	80
SCPP5-C2_SR	ATGTGGACATCTCTCCTGTGTCTTCTTCTGGCTGGTGCAGTATCCGCAGCACCACTTTCACCATTTTTCAATTATCTCCC	80

Sequence	81–160	
SCPP5_AM	GCACTATGGAAATCCAAGACCTGGACCTTCTGCACAGGGCACTAACGACTTCTTCTCTACCAATGGACAGCCTGCTTTCA	160
SCPP5_PN	GCATTATGGAAAACCA...GCAGGCCCCTCTGCACAGTTGTTAGTTTGCTTTTTTGGGTGCGGAAATTATCCTGCCTACG	157
SCPP5_CI	TCATTATGGCAGCCCGAAGCAGGTTGCTACAGAA......CATGGA......ATACCATCACAGCCACATCCTGGTCTGA	148
SCPP5_DR	TCATTATGGCAGCCCGAGGCAGGGGAATACTGGTGGCTTCCAGGGA......ATGCCATCACAGCCACATCCTGCTATGA	154
SCPP5_LW	TCATTATGGCAGCCCGAGGCAGGGGAATAATGGTGGCTTTCAGGGA......ATGCCATCACAGCAACATCCTGGTCTGA	151
SCPP5_PP	TCATTATGGCAGCCCGAAGCAGGGGAATAATGGTGGCTTTCAGGGA......ATACCATCACAA......CCTGGTCTGA	148
SCPP5-C1_CC	TCATTATGGCAACCCGAAGCAGGGAAATAATGGTGGCTTCCAGGGA......ATACCATCACAGCCACATCCTGGTCTGA	154
SCPP5-C2_CC	TCATTATGGCAACCCGAAGCAGGGGAATAATGGTGCTTTCCAGGGA......ATCCCATCACAGCCACATCCTGGTCTGA	154
SCPP5-C1_SA	TCATTATGGCAACCCGAAGCAGGGGAATAATGGTGGCTTCCAGGGA......ATACCAGCACAGCCACATCCTGGTCTGA	154
SCPP5-C2_SA	TCATTATGGCAACCCAAAGCAGGGGAATAATGGTGGCTTCCAGGGA......ATACCATCACAGCTACATCCTGGTCTGA	154
SCPP5-C1_SG	TCATTATGGCAACTCGAAGCAGGGGAATAATGGTGGCTTCCAGGGA......ATACCAGCACAGCCACATCCTGGTCTGA	154
SCPP5-C2_SG	TCATTATGGCAACCCAAAGCAGGGGAATAATGGTGGCTTCCAGGGA......ATACCATCACAGCCACATCCTGGTCTGA	154
SCPP5-C1_SR	TCATTATGGCAACCCGAAGCAGGGGAATAATGGTGGCTTCCAGGGA......ATACCAGCACAGCCACATCCTGGTCTGA	154
SCPP5-C2_SR	TCATTATGGCAACCCAAAGCAGGGGAATAATGGTGGCTTCCAGGGA......ATACCATCACAGCTACATCCTGGTCTGA	154

Sequence	161–240	
SCPP5_AM	ACGCCCCCATCAGTATGGAGATTGTCTTTCCTCCACAGTTCCAGGGATCTCCAGTAGGAGGAGCTGGTGCTGGACCTGCT	240
SCPP5_PN	CC...CCCATCAGTATGGAGATTGTGTTTCCTCCACAGTTCCCACGCACC...GCAGGGGGGGCTGGCGCCGGAGCATCT	231
SCPP5_CI	CTGCTCCAATCAGCATGGAAATCATTTTCCCACCGAGATTCCCTACTAATCCAGCAGGGGGAGCAGCTGGCACATCGTCA	228
SCPP5_DR	ATGCTCCGATCAGCATGGAAATCATTTTCCCTCCGCGATTCCCTGCTAATCCAGCAGGAGGAGCAGCGGGAACATCGTCA	234
SCPP5_LW	ACGCTCCAATCAGCATGGAAATCATTTTCCCACCGAGCTTCCCAACTAATCCAGCAGGGGGCGCAGCGGGCAAATCGTCA	231
SCPP5_PP	ACGCACCAATCAGCATGGAAATCATTTTCCCACCGAGATTCCCTACTAATCCAGCAGGGGGCAAAGCGGGCACATCGTCA	228
SCPP5-C1_CC	ACGCTCCAATCAGCATGGAAATCATTTTCCCTCCGCGATTCCCCGCTACTCCAGTAGGGGGAGCAGCTGGCGCATCGTCA	234
SCPP5-C2_CC	ATGCTCCAATCAGCATGGAAATTATTTTTCCTCCGCAATTCCCTGTCAGTCCGGTAGGGGGAGCAGCGGGCACATCGTCA	234
SCPP5-C1_SA	ACACTCCAATCAGCATGGAAATCATTTTCCCTCCGCGATTCCCTGCTACTCCA...GGAGGAGCAGCTGGCACATCGTCA	231
SCPP5-C2_SA	ATGCTCCAATCAGCATGGAAATTATTTTTCCTCCGCGATTCCCTGCTAGTCCAGTAGGGGGAGCAGCGGGCACATCGTCA	234
SCPP5-C1_SG	ATGCTCCAATCAGCATGGAAATCATTTTCCCTCCGCGATTCCCTGCTACTCCAGTAGGAGGAGCAGCTGGCGCATCGTCA	234
SCPP5-C2_SG	ATGCTCCAATCAGCATGGAAATTATTTTTCCTCCGCGATTCCCTGCTAGTCCAGTAGGGGGAGCAGCGGGCACATCGTCA	234
SCPP5-C1_SR	ACGGTCCATTCAGCATGGAAATCATTTTCCCTCCGCGATTCCCTGCTACTCCAGTAGGAGGAGCAGCTGGTACATCGGTG	234
SCPP5-C2_SR	ATGCTCCAATCAGCATGGAAATTATTTTTCCTCCGCGATTCCCTGCTAGTCCAGTAGGGGGAGCAGCGGGCACATCGTCA	234

Sequence	241–306	
SCPP5_AM	TTCCCATCCCAGGGCTTCATCAAATACTCTCTTCCTAAGGTTCCCGGCAGAAAGAGTGTGGAAATT	306
SCPP5_PN	TTTCCATCCCAGGCCTACATCAAATACTCTCTCCCCAAGGCTCCAGGTGCCCAGAGCGTGGAAGTT	297
SCPP5_CI	TTCCCAGCACAAGGGTTCATCAAGTACTCTCTCCCAAAGGCTCCCGGCAGAAAAAGCGTGGAAATC	294
SCPP5_DR	TTCCCCACACAAGCGTTCATCAAGTACTCGCTCCCCAAAGCTCCCGGCAGAAAAAGCGTTGAAATC	300
SCPP5_LW	TTCCCTACACAAGCGTTCATCAAGTACTCTCTCCCCAAGGCTCCGGGCAGAAAAAGCGTGGAAATT	297
SCPP5_PP	TTCCCTACACAAGCGTTCATCAAGTACTCTCTCCCCAAGGCTCCCGGCAGAAAAAGCGTGGAAATC	294
SCPP5-C1_CC	TTCCCTACACAAGCGTTCATCAAATACTCTCTCCCTAAAGTTCCCGGCAGAAAAAGCGTGGAAATC	300
SCPP5-C2_CC	TTCCCTACACAAGGGTTCATCAAGTACTCTCTCCCTAAAGCTCCCGGCAGAAAAAGCGTGGAAATC	300
SCPP5-C1_SA	CTGATGCAGAGTGTTGCTTTAGCCTTTGCAAATGTCTCATTAAATTGCAGAGATAAACATAATTTT	297
SCPP5-C2_SA	TTCCCTACACAAGGGTTCATCAAGTACTCTCTCCCTAAAGCTCCCGGCAGAAAAAGCGTGGAAATC	300
SCPP5-C1_SG	TTCCCTACACACGCGTTCATCAAATACTCTCTCCCTAAACTTCCCGGCAGAAAAAGCGTGGAAATC	300
SCPP5-C2_SG	TTCCCTACACAAGGGTTCATCAAGTACTCTCTCCCTAAAGCTCCCGGCAGAAAAAGCGTGGAAATC	300
SCPP5-C1_SR	AGTCAGTAAAGTTCGTTCATCAAATACTCTCTCCCTAAACTTCCCGGCAGAAAAAGCGTGGAAATC	300
SCPP5-C2_SR	TTCCCTACACAAGGGTTCATCAAGTACTCTCTCCCTAAAGCTCCCGGCAGAAAAAGCGTGGAAATC	300

X non conserved
X ≥55% conserved
X ≥85% conserved
