# Supplementary material for: A Genomic Survey of SCPP Family Genes in Fishes Provides Novel Insights into the Evolution of Fish Scales
Source: Int J Mol Sci. 2017 Nov 16;18(11):2432. doi: 10.3390/ijms18112432 (PMC5713400; doi:10.3390/ijms18112432)
Supplement: Supplementary file 1 [file Figure S9.fa93e10.cds.pdf › ijms-236743-supplementary/Figure S9.fa93e10.cds.pdf]

```
                5 10 15 20 25 30 35 40 45 50 55 60 65 70 75 80
conservation    !!!!!!!***!!!!**!*!!*!!!!!!!!**!!!!!!***!**!*!!*!!*!!*!!*!**!!!!!**!!!!**!*!!!!!
fa93e10˙AM      ATGAAAGCTGTGGCAATCTTCTTGTGTTTTGTGGCCTACACATTCGCTGCCCCAGCGCCCGACAGCGGCAGCCAGGAGCA   80
fa93e10˙CI      ATGAAAGCTGTGGCGTTGTTTATGTGTTTGCTGGGCTCCTCACTAGCTGCCCCTGCCCTTGACAGCGGCAGCAATGAGCA   80
fa93e10˙DR      ATGAAAGCTGTGGCGCTGTTGATGTGTTTGCTGGGCTCTTCACTAGCTGCCCCTGCCCCTGACAGTGGCAGTAATGAGCA   80
fa93e10˙LW      ATGAAAGCTGTGGCCTTGTTGATGTGTTTGCTGGGCTCCTCGCTAGCTGCACCTGCCCTCGACAGTGGCAGCAATGAGCA   80
fa93e10˙PN      ATGAAAGCTCTGGTGTTCTTGATGTGTTTTGTGGGCTGCACAGTAGCCGCCCCAGTGCCTGAAAGTGGGAGCCAAGAGCA   80
fa93e10˙PP      ATGAAAGCTGTGGCGTTGTTGATGTGTTTGCTGGGCTCATCGCTAGCAGCACCTGCCCCTGACAGCAGCAGCAATGAGCA   80
fa93e10-C1˙CC   ATGAAAGCAGTGGCGTTCTTGATGTGTTTGCTGGGCTCTTCACTTGCTGCCCCTGCCCCTGACAGTAGCAGTAATGAGCA   80
fa93e10-C2˙CC   ATGAAAGCTGTGGCATTCCTGATGTGTTTACTGGGCTCCTCAATAGCTGCCCCTGCCCCTGACAGTGACAGCAATGAGCA   80
fa93e10-C1˙SG   ATGAAAGTGCTGGCGTTCTTGATGTGTTTGCTGGGCTCTTCACTAGCTGCCCCTGCCCCTGACAGTGGCAGCAATGAGCA   80
fa93e10-C2˙SG   ATGAAAGCTGTGGCATTCTTGATGTGTTTGCTGGGCTCCTCACTAGCTGCCCCTGCCCCCGACAGTGGCAGCAATGAGCA   80
fa93e10-C1˙SA   ATGAAAGTGCTGGCGTTCTTGATGTGTTTGCTGGGCTCTTCACTAGCTGCCCCTGCCCCTGACAGTGGCAGCAATGAGCA   80
fa93e10-C2˙SA   ATGAAAGCTGTGGCATTCTTGATGTGTTTGCTGGGCTCCTCACTAGCGGCCCCTGCCCCTGACAGTGGCAGCAATGAGCA   80
fa93e10-C1˙SR   ATGAAAGTGCTGGCGTTCTTGATGTGTTTGCTGGGCTCTTCACTAGCTGCCCCTGCCCCTGACAGTGGCAGCAATGAGCA   80
fa93e10-C2˙SR   ATGAAAGCTGTGGCATTCTTGATGTGTTTGCTGGGCTCCTCACTAGCTGCCCCTGCCCCTGACAGTGGCAGCAATGAGCA   80

                85 90 95 100 105 110 115 120 125 130 135 140 145 150 155 160
conservation    !****!** !***!!!!!!!!!!!!!! *!!!!!*!!!!!!!!!!!!!!!!!*!!***!!*!!**!!! !!!!!!!!!**
fa93e10˙AM      GCAGATAGCTGCCCATGCTAACACAGCCCTGCATCTGATGGAACTCTACAGGATGTATGGCCACCTGCAGCAACAGGGTG   160
fa93e10˙CI      GGCAATTAATAGTCATGCTAACACAGCCCTGCAGTTGATGGAATTCTACAGAATATTAGGACAGCTACGGCAACAGGGTT   160
fa93e10˙DR      GGCAATTGCTAGTCATGCAAACACAGCTTTGCAGTTGATGGAGCTCTACAGAATGCTAGGACAGCTGCGGCAACAGGGAT   160
fa93e10˙LW      GGCAATTACTAGTCATGCTAACACAGCTCTGCAGTTGATGGAACTCTACAGAATGTTAGGACAGATGCAGCAACAGGGTT   160
fa93e10˙PN      GCAGATAGCGGCCCACGCTCACACAGCCCTACAGCTGATGGAACTCTACCGACTGTATGGCCACCTGCAGCATCAGGGCG   160
fa93e10˙PP      G...GTAGCTAGTCATGCTAACACAGCTGTGCAGTTGATGGAACTCAACAGATTGATAGCACAGATGCGGCAACAGGGTT   157
fa93e10-C1˙CC   GCAG...GCTGGTCATGCTAACACAGCTCTGCAGTTGATGGAACTCTACAGAATGTTAGGACATCTGCAGCAACAGGGAT   157
fa93e10-C2˙CC   GGCAATTATTAGTCATGCTAACACAGCACTGCAGTTGATGGAACTCTACAGAATGTTTGGACATCTGCGGCAACAGGGTT   160
fa93e10-C1˙SG   GGCAATTATTGGTCATGCTAACACAGCTCTGCAGTTGATGGAACTCTACAGAATGTTAGGACATCTGCAGCAACAGGGTT   160
fa93e10-C2˙SG   GGCAATTATTAGTCATGCTAACACAGCGCTGCAGTTGATGGAACTCTACAGAATGTTTGGACATCTGCGGCAACAGGGTT   160
fa93e10-C1˙SA   GGCAATTATTGGTCATGCTAACACAGCTCTGCAGTTGATGGAACTCTACAGAATGTTAGGACATCTGCAGCAACAGGGTT   160
fa93e10-C2˙SA   GGCAATTATTAGTCATGCTAACACAGCACTGCAGTTGATGGAACTCTACAGAATGTTTGGACATCTGCGGCAACAGGGGT   160
fa93e10-C1˙SR   GGCAATTACTGATCATGCTAACACAGCTCTGCAGTTGATGGAACTCTACAGAATGTTAGGACATCTGCAGCAACAGGGTT   160
fa93e10-C2˙SR   GGCAATTATTAGTCATGCTAACACAGCACTGCAGTTGATGGAACTCTACAGAATGTTTGGACATCTGCGGCAACAGGGTT   160

                165 170 175 180 185 190 195 200 205 210 215 220 225 230 235
conservation    !!!**!!*   !*!!!***!******!!**!*!**!*******     **!! **!***!! !**!!!!*!**!*!!!!!
fa93e10˙AM      TCGCAGCTCCTGCTCCAGCTCCAGTTCAGTACATCTACAAACCTATT...CAGAGCCCACCTAGCTCATGTTCCACCCAG   237
fa93e10˙CI      TTGGTGTT...GTTCCTACT.........GTACCTGTGGCACCCGCGGCACCCGCAGCACCCGCGCCTGTTGATACCCAG   228
fa93e10˙DR      ATGGTGCT...GTT...GCTGGTCCTCAACTGCCTGCCGCACCTGCAGCTCCCGTAGATCCTGCTCCTGTTGATACCCAG   234
fa93e10˙LW      TTGGTGCC...GTTCCT...GGTGCTCAGCTGCCCGCAGCACCTGTGGCACCCGCAGCACCAGCGCCTGTTGATACCCAG   234
fa93e10˙PN      TTGCAGCT......CCTGCTCCACTCCAGGTACAGGCTCAGCCTGCTCAAGCACCACAGCCGGTTCCTGTAGCTAATCAG   234
fa93e10˙PP      TTGGTGCC...GCTCCTAATGATGCTCAGCTGCCTGCAGCACCCGTGGCACCCGCAGCACCAGCGCCTGTTGATACCCAG   234
fa93e10-C1˙CC   TTGGTGCA...GTTCCTACTGGTGCTCAACTGCTTGCAGCACCTGCAGCGCCTGCAGCACCCGCACCTGTTGATGCCCAG   234
fa93e10-C2˙CC   TTGGTGCT...GTTCCTATTGGTGCTCAACTGCCTGCAGCACTCTCAGCGCCCGCAGCGCCCGCACCTGTTCATGCCCAG   237
fa93e10-C1˙SG   TTGGTGCT...GTTCCTACTGGTGCTCAACTGCCTGCAGCA.........CCCGCAGAACCTGCACCTGCTGATGCCCAG   228
fa93e10-C2˙SG   TTGGTGCT...GTTCCTACTGGTGCTCAACTGCCTGTATGTTAAAAATCACCTGCAGCGCCTGCACCTGTTGATGCCCAG   237
fa93e10-C1˙SA   TTGGTGCT...GTTCCTACTGGTGCTCAACTGCCTGCAGCACCTGCAGCACCCGTAGCACCCGCACCTGCTGATGCCCAG   237
fa93e10-C2˙SA   TTGGTGCT...GTTCCTACTGGTGTTCAACTGCCTGTACGTTAAAAATCACCTGCAGCGCCCGCACCTGTTGATGCCCAG   237
fa93e10-C1˙SR   TTGGTGCT...GTTCCTACTGGTGCTCAACTGCCTGCAGCA.........CCTGCAGCACCCGCACCTGCTGATGCCCAG   228
fa93e10-C2˙SR   TTGGTGCT...GTTCCTACTGGTGCTCAACTGCCTGTATGTTAAAAATCACCTGCAGCACCTGCACCTGTTGATGCCCAG   237

                240 245 250 255 260 265 270 275 280 285 290 295 300 305 310
conservation    !!*!!*!!!*!**!!   *!***!!!*!!*!!*!!**!*!!*!!!!!!****!*****!**!*!!***!!!!!!!!* !!
fa93e10˙AM      GCACAGGCTCAGTCT...GTTCAACCTCAGCAACCCTTCCTGTTTAACTACCCAACCCTGCCCAGAGGAGGAGATAACTC   314
fa93e10˙CI      CCTCAGGTTTCACCT...CAAGCACCACAACAGGGCTTTTTTTTCAACCCCGCTCTTTTTGCACCCAGAGGAGATGGGTC   305
fa93e10˙DR      CCTCAGGTTTCACCT...CAAGTACCTCAGCAGGGATTTTTCTTCAACCCTGCTGCTTTCGCGCCCACAGGAGACGGATC   311
fa93e10˙LW      CCTCAGGTTTCACCT...CAAGCACCACAGCAGGGCTTTTTTTTCAACCCCGCTCTTTTCGCACCCCGAGGAGATGGTTC   311
fa93e10˙PN      CAAGACGTACCGAGTTTAAACATATCAAGACAAGGATTCTTGGTCAACTACCCTGCATTGCCCCCTAGAGAAGATGACTC   314
fa93e10˙PP      CCTCAGGTTTCACCT...CAAGCTCCACAGCAGGGCTTTTTTTTCAACCCCGCTCTTTTCGCACCCCAAGGAGATGGGTC   311
fa93e10-C1˙CC   CCTCAAGTTTCACCT...CAAGCACCACAGCAGGGCTTTTTTTTCAACCCCGCTCTTTTCGCACCCACAGGAGATGGATC   311
fa93e10-C2˙CC   CCTCAGGTTTCACCT...CAAGCACCACAGCAGGGCATTTTCTTCAAC...............CCCACAGGAGATGAATC   299
fa93e10-C1˙SG   CCTCAAGTTTCACCT...CAAGCACCACAACAGGGCTTTTTTTTCAACCCCGCTCTTTTCGCACCCACAGGAGATGGATC   305
fa93e10-C2˙SG   CCTCAGGTTTCACCT...CAAGCACCACAGCAGGGCATTTTTTTCAACCCCGCTCTTTTCGCACCCACAGGAGATGGGTC   314
fa93e10-C1˙SA   CCTCAAGTTTCACCT...CAAGCACCACAGCAGGGCTTTTTTTTCAACCCCGCTCTTTTCGCACCTACAGGAGATGGATC   314
fa93e10-C2˙SA   CCTCAGGTTTCACCT...CAAGCACCACAGCAGGGCATTTTTTTCAACCCCGCTCTTTTCGCACCCACAGGAGATGGGTC   314
fa93e10-C1˙SR   CCTCAAGTTTCACCT...CAAGCACCACAGCAGGGCTTTTTTTTCAACCCCGCTCTTTTCGCACCCACAGGCGATGGATC   305
fa93e10-C2˙SR   CCTCAGGTTTCACCT...CAAGCACCACAGCAGGGCATTTTTTTCAACCCCGCTCTTTTCGCACCCACAGGAGATGGGTC   314

                315 320 325 330 335 340 345 350 355 360 365 370 375 380 385 390
conservation    *!!!!!!!!!!*!** !!*!!*!*!**!!!*   !!!!!*!!*!!*!!*!!!!*!               ***!!*!!**
fa93e10˙AM      TGATGAGGAAGCACAGCCTAATTACGCTGCA...TTTTACCCTCCCCACGGCTTCCCAGCTGTACCAGCAGCACCGGCGG   391
fa93e10˙CI      AGATGAGGAAGGAGCACCACAGTTTCGTGGG...TTTTACCCTCCCTACGGCTAC...............CAGCCTGGCC   367
fa93e10˙DR      TGATGAGGAAGTTGCTCCACAGTATCGTGGA...TTTTACCCACACTATGGTTACCAC............CAGCCTGCCC   376
fa93e10˙LW      AGATGAGGAAGGAGCACCACTGTATCGTGGG...TTTTACCCTCCCTACGGCTAC...............CAGCCTGCCC   373
fa93e10˙PN      TGATGAGGAAGCACAGCCCCAACATGTTGGTGCATTCTACCCACCGTATGGCTACCCAGCTGTACCTGCAGCCCCCGCAG   394
fa93e10˙PP      AGATGAGGAAGGAGCACCACAGTTTCGTGGG...TTTTACCCTCCCTACGGCTTC...............CAGCCTGCCC   373
fa93e10-C1˙CC   AGATGAGGAAGGAACTCCACAGTTTCGTGGG...TTTTACCCTCCCTACGGCTAC...............CAGCCTGCCC   373
fa93e10-C2˙CC   AGATGAGGAAGGAGCACCGCAGTTTCGTGGG...TTTTATCCTCCCTATGGCTAC...............CAGCCTGCCC   361
fa93e10-C1˙SG   AGATGAGGAAGGAGTTCCACAGTTTCGTGGG...TTTTACCCTCCCTACGGCTAC...............CAGCCTGCCC   367
fa93e10-C2˙SG   AGATGAGGAAGGAGCACCGCAGTTTCGTGGG...CTTTATCCTCCTTACGGCTAC...............CAGCCTGCCC   376
fa93e10-C1˙SA   AGATGAGGAAGGAGCTCCACAGTTTCGTGGG...TTTTACCCTCCCTACGGCTAC...............CAGCCTGCCC   376
fa93e10-C2˙SA   AGATGAGGAAGGAGCACCGCAGTTTCGTGGG...TTTTATCCTCCTTACGGCTAC...............CAGCCTGCCC   376
fa93e10-C1˙SR   AGATGAGGAAGGAGCTCCACAGTTTCGTGGG...TTTTACCCTCCCTACGGCTAC...............CAGCCTGCCC   367
fa93e10-C2˙SR   AGATGAGGAAGGAGCACCGCAGTTTCGTGGG...TTTTATCCTCCCTACGGCTAC...............CAGCCTGCCC   376
```

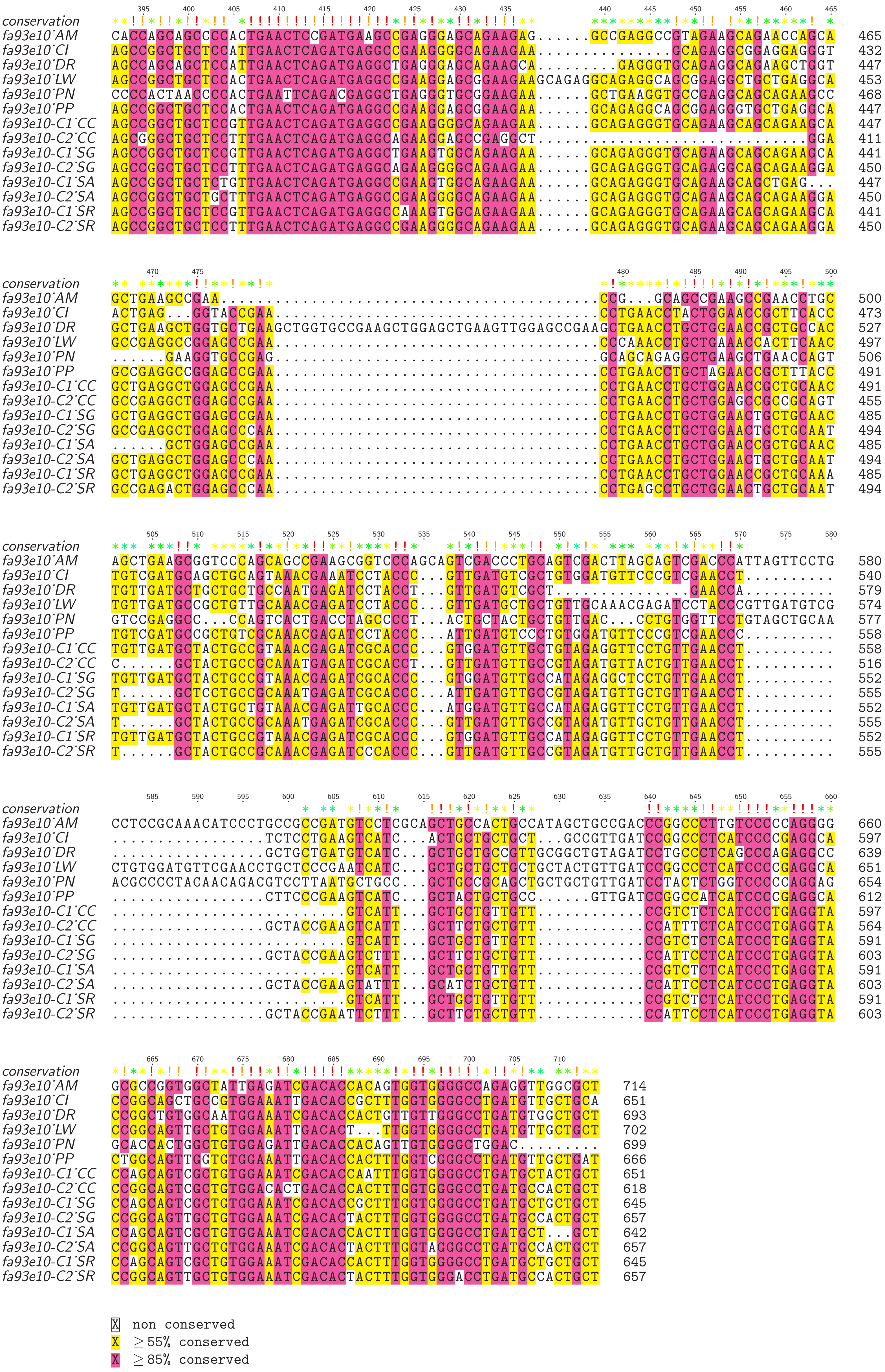
X non conserved
X ≥55% conserved
X ≥85% conserved
