# Supplementary material for: A Genomic Survey of SCPP Family Genes in Fishes Provides Novel Insights into the Evolution of Fish Scales
Source: Int J Mol Sci. 2017 Nov 16;18(11):2432. doi: 10.3390/ijms18112432 (PMC5713400; doi:10.3390/ijms18112432)
Supplement: Supplementary file 1 [file ijms-18-02432-s001.zip › ijms-236743-supplementary/Figure S2.SPP1.cds.pdf]

```
                  5   10   15   20   25   30   35   40   45   50   55   60   65   70   75   80
conservation  *********** ****************** ******************* ***************** ***********
SPP1˙AM       ATGAAGGCCGCCATTGTTTTCGTGCTCCTCTTTGCCATAGTCTACTGTCGTCCTCTGAAGCGTTCAGTGAGCAGCTCAGA   80
SPP1˙CI       ATGAAGACTCTTATCGTTTTAACGCTCCTCGTTGCCACAGTCTTCTGCCTACCTGTCAAGCGCTCAGCTAGCAGTTCAGA   80
SPP1˙DR       ATGAAATCTATTATTGTTTTAACACTCCTCGTCGCCACAGTCTTCTGTGTACCTGTGAAGCGCTCAGCAAGCAGTTCAGA   80
SPP1˙IP       ATGAAGGCTGTCATTGTTTTCGTGCTCCTTTTCGCAGCGGTCTATTGTCACCCTGTGAAACGTTCAGCCAGCAGCTCAGA   80
SPP1˙LW       ATGAAGACCGTTATCGTTTTAACCCTCCTCATCGCCACAGTCTTCTGCCTGCCTGTCAAGCGCTCAGCCAGCAGTTCAGA   80
SPP1˙PN       ATGAAGGCCGTCATGATTTTCGGGCTCCTCTTCGCTGTAGTCTACTGCCGTCCTGTAAAACGCTCAGCAAGCAGCTCAGA   80
SPP1˙PP       ATGAAGACTGTTATCGTTTTAACGCTTCTCATCGCCACAGTCTTCTGCCTACCTGTCAAGCGCTCAGCCAGCAGTTCAGA   80
SPP1-C1˙CC    ATGAAGACTGTCATCGTTTTAACGCTCCTCATCGCCACAGTTTTCTGTCTACCTGTGAAGCGCTCAGCTAGCAGTTCAGA   80
SPP1-C2˙CC    ATGAAGACTGTTTTCGTTTTAACGCTCCTCATCGCCACAGTCTTCTGTCTACCTGTGAAGCGCTCAGCTAGCAGTTCAGA   80
SPP1-C1˙SG    ATGAAGACTGTCATCGTTTTAACGCTCCTCATCGCCACAGTTTTCTGTCTACCTGTGAAGCGCTCAGCTAGCAGCTCAGA   80
SPP1-C2˙SG    ATGAAGACTGTTATCGTTTTAACGCTCCTCATCGCCACAGTCTTCTGTCTGCCTGTGAAGCGCTCAGCTAGCAGTTCAGA   80
SPP1-C1˙SR    ATGAAGACTGTCATCGTTTTAACGCTCCTCATCGCCACAGTTTTCTGTCTACCTGTGAAGCGCTCAGCTAGCAGTTCAGA   80
SPP1-C2˙SR    ................................................................................    0
SPP1-C1˙SA    ATGAAGACTGTCATCGTTTTAACGCTCCTCATCGCCACAGTTTTCTGTCTACCTGTGAAGCGCTCAGCTAGCAGTTCAGA   80
SPP1-C2˙SA    ATGAAGACTGTTATCGTTTTAACACTCCTCATCGCCACAGTCTTCTGTCTGCCTGTGAAGCGCTCGGCTAGCAGTTCAGA   80

                 85   90   95  100  105  110  115  120  125  130  135  140  145  150  155
conservation  *************** ** *  ** ***** *************** * ************** ******* *********
SPP1˙AM       GAGTTCTGAGGAACAAGCCGCTGCCAAACCGGCGCCCCCAATTCTGCGGAAAGCCCCTGCAAAAGCAGTGCAGGCA....  156
SPP1˙CI       GAGCTCAGAAGAGCTTGTTGCTGTTCAACGGCCACCTCCTATTCTCCGAAAAGCTGCTGCTAATATGTTTGAGGCAGAAC  160
SPP1˙DR       GAGCTCAGAAGAGCTTGTAATTGTTCAACGGCCACCTCCTATTCTTCGAAAAGCTGCTGCCAATGTGTTCCAGGTAGAGC  160
SPP1˙IP       GAGTGCTGACCTGCAGCCTGTTGATACTCAGAGC......ATCAATGGAAAAAGAATCACAGTAGCCATATTCACAAAGC  154
SPP1˙LW       GAGCTCAGAAGAAATGATTGCTGTTCAACGGCCACCTCCTCTTCTACAAAAAGCTGCTGCTGATATGTTCCAGGCAGAAT  160
SPP1˙PN       GAGCTCTGAGGAACAGGCAGTTGCCAAACTGCCACTCCCAGTTCTTGATAAAGGCCCTGCAGATCCACTAAAGGCAGCAC  160
SPP1˙PP       GAGCTCAGAAGAAATGATTGCCGTTCAACGGCCACCTCCTATTCTA...AAAGCTGCTGCTGATATGTTCCAGGCAGAAC  157
SPP1-C1˙CC    GAGCTCAGAAGAACTTGTGGTTGCTCAACAACCAACTCCTATTCTACGAAAAGCTGCTGCTGTTATGTTCAAGGCAGAAC  160
SPP1-C2˙CC    GAGCTCAGAAGAACTTGTGACTGTTCAACAACCACCTCCTATTCTAGAAAAAGCTGCTGCTGTTATGTACAAGGCAGAAC  160
SPP1-C1˙SG    GAGCTCAGAAGAACTTGTGGTTGCTCAACAACCACCTCCTATTCTACGAAAAGCTGCTGCTGTTATGTTCAAGGCAGAAC  160
SPP1-C2˙SG    GAGCTCAGAAGAACTTGTGGTTGTTCAACGGCCACCTCCTATTCTGCAAAAAGCTGCTGCTGTTATGTTCAAGGCAGAAC  160
SPP1-C1˙SR    GAGCTCAGAAGAACTTGTGGTTGCTCAACGGCCACCTCCTATTCTACGAAAAGCTGCTGCTGTTATGTTCAAGGCAGAAC  160
SPP1-C2˙SR    ................................................................................    0
SPP1-C1˙SA    GAGCTCAGAAGAACTTGTGGTTGCTCAACGGCCACCTCCTATTCTACGAAAAGCTGCTGCTGTTATGTTCAAGGCAGAAC  160
SPP1-C2˙SA    GAGCTCAGAAGAACTTGTGGTTGTTCAACGGCCACCTCCTATTCTGCAAAAAGCTGCTGCT...ATGTTCAAGGCAGAAC  157

                160  165  170  175  180  185  190  195  200  205  210  215  220  225  230
conservation  ******!****** ***** *!****************   *!******!******* ****** ***************
SPP1˙AM       ..GTAGAGGTTGTACCTGCTCAGGCTGTTAAAGCAGCTCCAGAAGCTCCAGCAGATCCAGAAGCAGCAGCAGCGGCAGCA  234
SPP1˙CI       CTACACAGACCACGCCAACAGAATCTAATGAAAGCACA...GACAGTGCAGATGACACTGAGGAGGCATATGAGGAATCT  237
SPP1˙DR       CTACACAGACCACGCCAACAGAATCGAATGAAAGCACA...GACAGCGCAGATGACACTGAGGAGGCAGATGAGGAATCT  237
SPP1˙IP       CTACCTAATTTCAAGTGTTTTATAATTACTACTCGGCA...CATACTGTATTGAAAGTCACTGCGGTTGCTGATGTTAGC  231
SPP1˙LW       CTACACAGACCACACCAGCAGATTCCAATGAAAGCACA...GACAGTGAAGATGACACTGAGGAGGCAGATGAGAAATCT  237
SPP1˙PN       CTGCACAGGACAAAAACACAGAATCAGACACAGAGGGA...GACAGTGCAGACAGCGCAGACACGCAAGACACGCAAGAC  237
SPP1˙PP       CTACACAGACCACACCAGCAGATTCCAATGAAAGCACA...GACAGTGAAGATGACACTGAGGAGGAAGATGAGGAATCT  234
SPP1-C1˙CC    CTACACAGTCCACGCCAGGAGAATCCAATGAAAGCACA...GACAGTGCAGATGACAGTGAGGGTGCAGATGAGGAATCT  237
SPP1-C2˙CC    CCACACAGACCACACCAGTAGATTCCAATGAAAGCACA...GACAGTGCAGATGACAGTGAGGATGCAGATGAGGAATCT  237
SPP1-C1˙SG    CTACACAGACCACGCCAGCTGAATCCAATGAAAGCACA...GACAGTGCAGATGACAGTGAGGATGCAGATGAGGAATCT  237
SPP1-C2˙SG    CTACACAGACCACGCCAGCTGAATCCAATGAAAGCACA...GACAGTGCAGATGACAGTGAGGATGCAGATGAGGAATCT  237
SPP1-C1˙SR    CTACACAGACCACGCCAGCTGAATCCAATGAAAGCACA...GAAAGTGCAGATGACAGTGAGGATGCAGATGAGGAATCT  237
SPP1-C2˙SR    .....CAGACCACGCCAGCTGAATCCAATGAAAGCACA...GACAGTTCAGATGACAGTGAGGATGCAGATGAGGAATCT   72
SPP1-C1˙SA    CTACACAGACCACGCCAGCTGAATCCAATGAAAGCACA...GACAGTGCAGATGACAGTGAGGATGCAGATGAGGAATCT  237
SPP1-C2˙SA    CTACACAGACCACGCCAGTAGAATCCAATGAAAGCACA...GACAGTGCAGATGACAGTGAGGATGCAGATGAGGAATCT  234

              235  240  245  250  255  260  265  270  275  280  285  290  295  300  305  310
conservation   *****!******* *!* * ****! ** ***!!***!**!!!***!   *!****!!*!!* !** ** ****** **
SPP1˙AM       TCATCGGATGAGGACGACTCAGATGAAGCTGAGGACTCCAGTGAGAGTGCTGAGTCTGGAGAAACTGAGGCCACCACCAC  314
SPP1˙CI       GAAACAGATGAGAACAAAGAGGAGAACGACACTGATTCCAGTGAAAGT...GAATCTGGAGAAACTGAGACCACCGCAGT  314
SPP1˙DR       GAAACAGATGAGAAGGAAGAGGAGAATGAGACTGACTCCAGTGAGAGT...GAATCTGGAGAATCTGATACCACCATCAT  314
SPP1˙IP       TCTGAAGAGGATGATGATGATGATGATGACAGTGACAGCGATGAGTCTGATGAGAATGGCGAGCCCACCAGTTCT.....  306
SPP1˙LW       GAAACAGATGAGGATGAG...GAGAATGAGACTGACTCCAGTGAGAGT...AATTCTGGAGAAACTCAGAGCACCGTAGT  311
SPP1˙PN       ACTATAGATTTGTGCCAGGATTGGGACACAGATGACTCCAGTGAGAGTGCTGAATCAGGAGAGACTGAGGCCACTACCGT  317
SPP1˙PP       AAAACAGATGAGGATGAG...GAGAACGAGACTGACTCCAGTGAGAGT...AATTCTGGGGAAACTCAGAGCACCGTCGT  308
SPP1-C1˙CC    GAAATGGATGAGAACGAAAAGGAGAACGATACTGACTCCAGTGAGAGT...GAATCTGGAGAACCTGCGACCACCGTTGT  314
SPP1-C2˙CC    GAAACAGATGAGAAGGAAGATGAGAATGATACTGATACCAGTGAGAGT...GAATCTGGAGAAACTGCGATCACCGTTGT  314
SPP1-C1˙SG    AAAACAGTTGAGAACGAAAAGGAGAACGACACTGACTCCAGTGAGAGT...GAATCTGGAGAATCTGCGACCATCGTTGT  314
SPP1-C2˙SG    GAAACAGATGAGAGGGAAGATGAGAATGATACTGATTCCAGTGAGAGT...GAATCTGGAGAATCTGCGATCACCGTTGT  314
SPP1-C1˙SR    GAAACAGTTGAGAACGAAAAGGAGAACGATACTGACTCCAGTGAGAGT...GAATCTGGAGAATCTGCGACCGCCGTTGT  314
SPP1-C2˙SR    GAAACAGATGAGAGGGAAGATGAGAATGATACTGATACCAGTGAGAGT...GAATCTGGAGAATCTGCGATCACCGTTGT  149
SPP1-C1˙SA    AAAACAGCTGAGAATGAAAAGGAGAACGATACTGACTCCAGTGAGAGT...GAATCTGGAGAATCTGCGACCACCGTTGT  314
SPP1-C2˙SA    GAAACAGATGAGAGGGAAGATGAGAATGATACTGATTCCAGTGAGAGT...GAATCTGGAGAATCTGCGATCACCGTTGT  311

              315  320  325  330  335  340  345  350  355  360  365  370  375  380  385  390
conservation  *   !! ***!**** !! *!!*! !!****!!**!*!!**!****!!*!!*!!!!!!!***!*!*!!**!**!!*!!!!
SPP1˙AM       CCCACCTTTCACCGAGGAGCCCACCCTGGGCCCTATTATTAACGACGGCCGCGGAGACAACATGGGCTACCCCAGCGACT  394
SPP1˙CI       C...CCATCCACAGTAGAGCCCTCACTGGACCCCATCATCAACACAGGCCGGGGAGACAGCATGGGCTACCCTAGCGACT  391
SPP1˙DR       C...CCAGTCACAGTCGATCCCACGCTGGGTCCCATTATCAACACAGGCCGGGGAGACAGTTTGGGCTACCCTAGCGACT  391
SPP1˙IP       ....CCTCCTATCATAGAGACCACTCTGATCCCTGTTATTAAT...GGCCGTGGAGACAGCATTGGCTTCCAGAGTGACT  379
SPP1˙LW       C...CCATCCACAGTCGAGCCCTCGCTGGACCCCATCATCAACACAGGCCGGGGAGACAGCATGGGCTACCCCAGCGACT  388
SPP1˙PN       CCCACCTGTAACCGTGGAGCCCACCCTGGACCCTATTATTGACAACGGTCGTGGAGACAGCATGGGCTACCCCAGCGACT  397
SPP1˙PP       C...CCATCCACAGTCGAGCCCTCGCTGGACCCCATTATCCACACAGGCCGGGGAGACAACATGGGCTACCCAAGCGACT  385
SPP1-C1˙CC    C...CCATTCACAGTGGAACCCCCACTGGACCCCATCATCAACACAGGCCGGGGAGACAACATGGACTATCCTAGCGACT  391
SPP1-C2˙CC    C...CCCTCCACAGTAGAACCCTCACTAGATCCCATCATCAACACAGGCCGGGGAGACAACATGGGCTACCCTAGCGACT  391
SPP1-C1˙SG    C...CCCTCCACAGTGGAACCCTCACTGGACCCCATCATCAACACAGGCCGGGGAGACAGCATGGGCTATCCTAGCGACT  391
SPP1-C2˙SG    C...CCCTCCACAGTAGAACCCTCACTGGACCCCATCATCAACACAGGCCGGGGAGACAGCATGGGCTACCCTAGCGACT  391
SPP1-C1˙SR    C...CCCTCCACAGTGGAACCCTCACTGGACCCCATCATCAACACTGGCCGGGGAGACAGCATGGGCTATCCTAGCGACT  391
SPP1-C2˙SR    C...CCCTCCACAGTAGAACCCTCACTGGACCCCATCATCAACACAGGCCGGGGAGACAGCATGGGCTACCCTAGCGACT  226
SPP1-C1˙SA    C...CCCTCCACAGTGGAACCCTCACTGGACCCCATCATCAACACAGGCCGGGGAGACAGCATGGGCTATCCTAGCGACT  391
SPP1-C2˙SA    C...CCCTCCACAGTAGAACCCTCACTGGACCCCATCATCAACACAGGCCGGGGAGACAGCATGGGCTACCCTAGCGACT  388
```

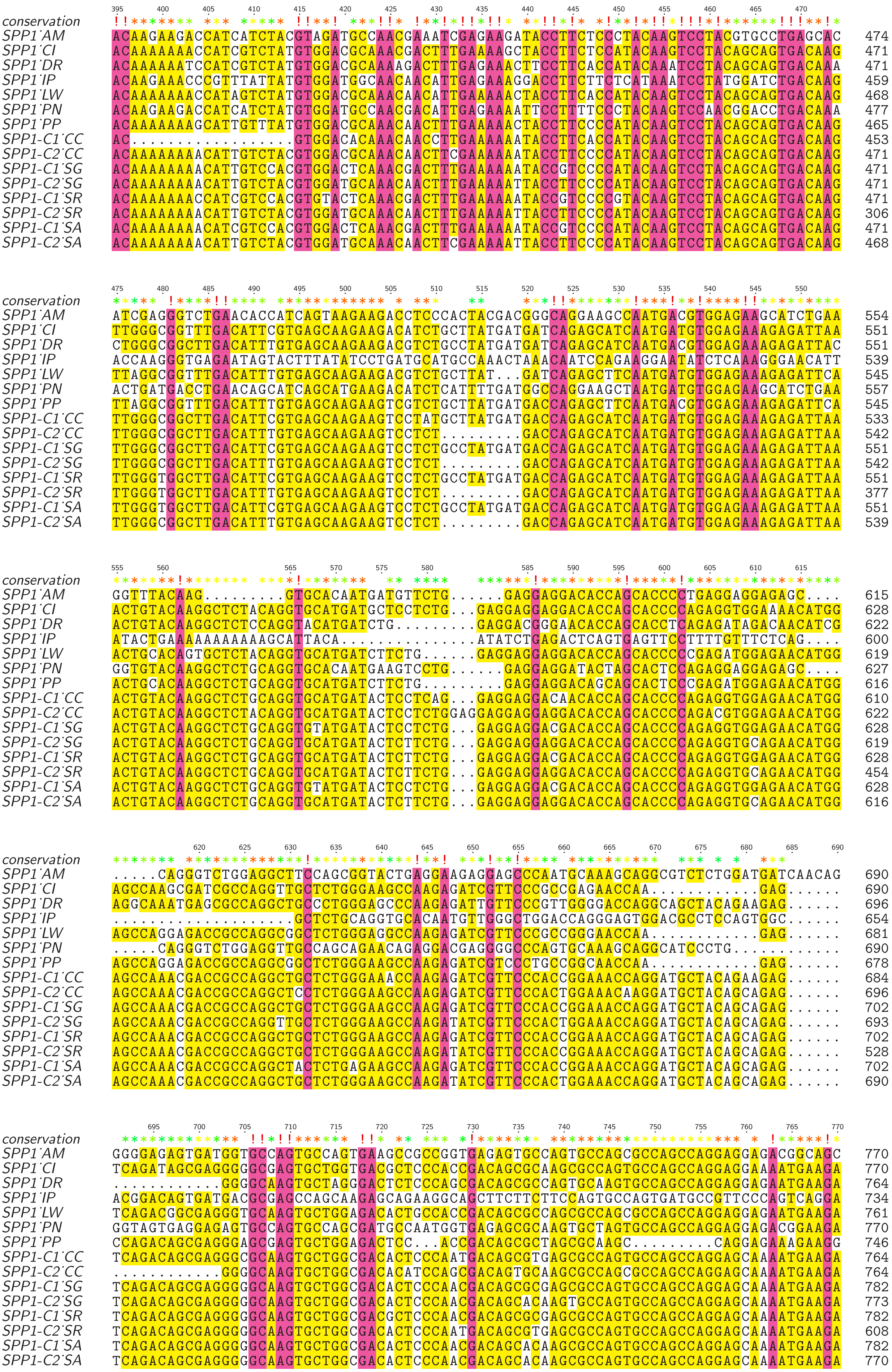

conservation
SPP1 AM 474
SPP1 CI 471
SPP1 DR 471
SPP1 IP 459
SPP1 LW 468
SPP1 PN 477
SPP1 PP 465
SPP1-C1 CC 453
SPP1-C2 CC 471
SPP1-C1 SG 471
SPP1-C2 SG 471
SPP1-C1 SR 471
SPP1-C2 SR 306
SPP1-C1 SA 471
SPP1-C2 SA 468
conservation
SPP1 AM 554
SPP1 CI 551
SPP1 DR 551
SPP1 IP 539
SPP1 LW 545
SPP1 PN 557
SPP1 PP 545
SPP1-C1 CC 533
SPP1-C2 CC 542
SPP1-C1 SG 551
SPP1-C2 SG 542
SPP1-C1 SR 551
SPP1-C2 SR 377
SPP1-C1 SA 551
SPP1-C2 SA 539
conservation
SPP1 AM 615
SPP1 CI 628
SPP1 DR 622
SPP1 IP 600
SPP1 LW 619
SPP1 PN 627
SPP1 PP 616
SPP1-C1 CC 610
SPP1-C2 CC 622
SPP1-C1 SG 628
SPP1-C2 SG 619
SPP1-C1 SR 628
SPP1-C2 SR 454
SPP1-C1 SA 628
SPP1-C2 SA 616
conservation
SPP1 AM 690
SPP1 CI 690
SPP1 DR 696
SPP1 IP 654
SPP1 LW 681
SPP1 PN 690
SPP1 PP 678
SPP1-C1 CC 684
SPP1-C2 CC 696
SPP1-C1 SG 702
SPP1-C2 SG 693
SPP1-C1 SR 702
SPP1-C2 SR 528
SPP1-C1 SA 702
SPP1-C2 SA 690
conservation
SPP1 AM 770
SPP1 CI 770
SPP1 DR 764
SPP1 IP 734
SPP1 LW 761
SPP1 PN 770
SPP1 PP 746
SPP1-C1 CC 764
SPP1-C2 CC 764
SPP1-C1 SG 782
SPP1-C2 SG 773
SPP1-C1 SR 782
SPP1-C2 SR 608
SPP1-C1 SA 782
SPP1-C2 SA 770

```
                  775  780  785  790  795  800  805  810  815  820  825  830  835  840  845
conservation   *******!!******** ****!!*!!*!!***!!!**!**! *!*****!**!*!!******* **************
SPP1˙AM        G...TCCGACAGCAGCCAAAGCAGCGAGGAGGCCACCGCCACCCCCGGTGCCGCCGACGGCGAGAGCGACTCCTCTCAG.   846
SPP1˙CI        GGAGTCAGATAGCAGCCCGAGCAGCGAGGATACCACAACCACGCCTGGAGCTGCAGACAGCGATGAAGACTCCTCACAG.   849
SPP1˙DR        AGAGTCGGAT............AGTGAGGAGACCACATCGACGCCGGGAGCTGCAGACAGCGAGTCCAACTCCTCTCAGA   832
SPP1˙IP        GAGTGAGGAGGACAGCCAGAGCAGTGAGGAGAACACAACCGCGGCTACCGCGACCGACAGC......AACTCCTCCGAG.   807
SPP1˙LW        GCAGTCAGAT.........AGCAGCGAGGAGACCACAGCCACCCCTGGAGCTGCAGACAGCGAGAGCGACTCCTCACAG.   831
SPP1˙PN        G...TCTGACAGCAGCAAGAGCAGCGAGGAGGCCACTGCCACCCCTGGGGCTGCAGACACAGAGAATGACTCCTCTCAG.   846
SPP1˙PP        GGAGTCGGAT.........AGCAGCGAGGAGACCACAGCCACCCCCGGAGCTGCAGACAGCGACAGCGACTCCTCACAG.   816
SPP1-C1˙CC     GGAGTCAGACAGCAGCCCGAGCAGCGAGGAGACCACAGCCACACCTGGAGCTGCAGACAGAGAGGACGACTCCTCACAG.   843
SPP1-C2˙CC     GGAGTCAGATAACAGCCTGAGCAGCGAGGAGACCACAGCCACACCTGGAGCTGCAGACAGCGAGGAG.............   831
SPP1-C1˙SG     GGAGTCAGATAGCAGCCCGAGCAGTGAAGAGACCACAGCCACGCCTGGAGCTGCAGACGGTGAGGACGACTCCTCACAG.   861
SPP1-C2˙SG     GGAGTCAGATAGCAGCCCGAGCAGCGAGGAGACCACAGCCACGCCTGGAGCTGCAGACAGCGAGGACGACTCCTCACAG.   852
SPP1-C1˙SR     GGAGTCAGACAGCAGCCCGAGCAGCGAGGAGACCACAGCCACGCCTGGAGCTGCAGACAGCGAGGACGACTCCTCACAG.   861
SPP1-C2˙SR     GGAGTCAGATAGCAGCCCGAGCAGCGAGGAGACCACAGCCACACCTGGACCTGCAGACAGCGAGGACGACTCCTCACAG.   687
SPP1-C1˙SA     GGAGTCAGATAGCAGCCCGAGCAGCGAGGAGACCACAGCCACGCCTGGAGCTGCAGAGAGCGAGGACAACTCCTCACAG.   861
SPP1-C2˙SA     GGAGTCAGATAGCAGCCCGAGCAGCGAGGAGACCACAGCCACGCCTGGAGCTGCAGACAGCGAGGACGACTCCTCACAG.   849

                  850  855  860  865  870  875  880  885  890  895  900  905  910  915
conservation           !!!***!!*!!***!!!!****!**!** *!!********* ************      *** ** * ** 
SPP1˙AM        ........AGCTCCGAGAGCCTGGAGGACGACTCCGCCGAGCAGACAGTGGAGGCAACGTCCGACATCCCTGACGTCATC   918
SPP1˙CI        ........AGCACAGAAAGCCAGGAGAGCGATTCGGATGAGGAGACCACCCAGACCACCGAG......GCTTCCATCGTA   915
SPP1˙DR        GCACAGAAAGCACAGAAAGCCAGGAGAGCGACTCAGATGAGGAGACCACTCAGACCACTGAG......GCCTCCATCATC   906
SPP1˙IP        ........AGCGATGAGAGTGAGGAGACCAACACAGCGGAT.......................................   840
SPP1˙LW        ........AGCACAGAAAGCCAGGAGAGCGATTCGGCTGAGGAGAACACACAGACCACCGAG......GCTTCCATCATA   897
SPP1˙PN        ........AGCTCTGAGAGTCAGGAGGATGACTCCGCCGAGCAGGCCATGGAAACCACGACCGATGTCCCTGACGTCATA   918
SPP1˙PP        ........AGCACAGAAAGCCAGGAGAGCGATTCGGATGAGCAGAACACACAGACCACCGAG......GCTTCCATCATA   882
SPP1-C1˙CC     ........AGCACAGAGAGCCAGGAGAGCGATTCGAGTGAGGATACCACCCAGACCACCGAC......GCTACTGTTATC   909
SPP1-C2˙CC     ........AGCACAGAGAGCCAGGAGAGTGATTCGAGTGAGGGGACCACCCAGACCACCGAC......CCTACTATTATC   897
SPP1-C1˙SG     ........AGCACAGAGAGCCAGGAGAGCGATTCGGATGAGGAGACCATCCAGACCACCGAC......GCTACTGTTATC   927
SPP1-C2˙SG     ........AGCACAGAGAGCCAGGAGAGCGATTCGGATGAGGAGACCACCCAGACCACCGAC......GCTACCGTTATC   918
SPP1-C1˙SR     ........AGCACAGAGAGCCAGGAGAGCGATTCGGATGAGGAGACCACCCAGACCACCGAC......GCTACCGTTATC   927
SPP1-C2˙SR     ........AGCACAGAGAGCCAGGAGAGCGATTCGGATGAGGAGACCACCCAGACCACCGAC......GCTACCGTTATC   753
SPP1-C1˙SA     ........AGCACAGAGAGCCAGGAGAGCGACTCGGATGAGGAGACCACCCAGACCACCGAC......GCTACTGTTATC   927
SPP1-C2˙SA     ........AGCACAGAGAGCCAGGAGAGTGATTCGGATGAGGAGACCACCCAGACCACCGAC......GCTACCGTTATG   915

                920
conservation   *
SPP1˙AM        ATC   921
SPP1˙CI        ACC   918
SPP1˙DR        ATC   909
SPP1˙IP        ...   840
SPP1˙LW        AGC   900
SPP1˙PN        ATC   921
SPP1˙PP        AAC   885
SPP1-C1˙CC     AAA   912
SPP1-C2˙CC     ACA   900
SPP1-C1˙SG     ATA   930
SPP1-C2˙SG     ATA   921
SPP1-C1˙SR     ATA   930
SPP1-C2˙SR     ATA   756
SPP1-C1˙SA     ATA   930
SPP1-C2˙SA     ATA   918
```

X non conserved

X ≥ 55% conserved

X ≥ 85% conserved
