# Supplementary material for: A Genomic Survey of SCPP Family Genes in Fishes Provides Novel Insights into the Evolution of Fish Scales
Source: Int J Mol Sci. 2017 Nov 16;18(11):2432. doi: 10.3390/ijms18112432 (PMC5713400; doi:10.3390/ijms18112432)
Supplement: Supplementary file 1 [file ijms-18-02432-s001.zip › ijms-236743-supplementary/Figure S3.SCPP9.cds.pdf]

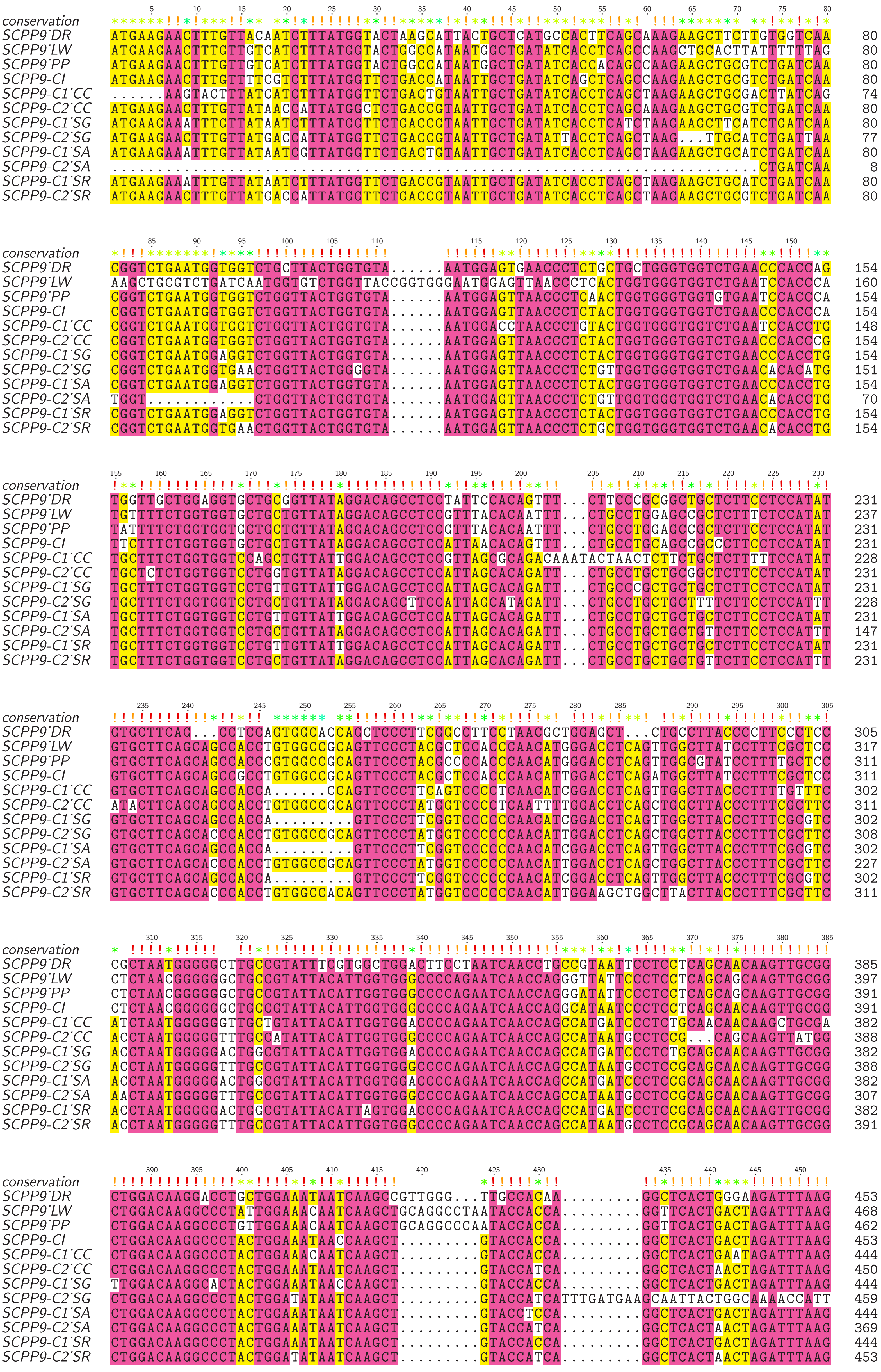
conservation
SCPP9 DR 80 154 231 305 385 453
SCPP9 LW 80 160 237 317 397 468
SCPP9 PP 80 154 231 311 391 462
SCPP9-CI 80 154 231 311 391 453
SCPP9-C1 CC 74 148 228 302 382 444
SCPP9-C2 CC 80 154 231 311 388 450
SCPP9-C1 SG 80 154 231 302 382 444
SCPP9-C2 SG 77 151 228 308 388 459
SCPP9-C1 SA 80 154 231 302 382 444
SCPP9-C2 SA 8 70 147 227 307 369
SCPP9-C1 SR 80 154 231 302 382 444
SCPP9-C2 SR 80 154 231 311 391 453

```
conservation   !!!!!*!!*!!!!!!!!!!!!!!****!!!***!!!!!!!***!!!!*!!!!!*!!!!!*!!!*!!!*!!!**!!!!!*!
SCPP9˙DR       CGATCATTTATCAGAAGGACCACAGCAAGACCACCTGTCTCAGTCACCCAGATGCCTGCTCAGCTCAGCCCTACTGTGTC   533
SCPP9˙LW       CGCTCCTTCCTCAGAAGGACCACA...AGACCACCTGTCTCCATCACCCAGATCCCAGCTCAGGTCAGCCCAACTGTGTC   545
SCPP9˙PP       CGCTCCTTCCTCAGAAGGACCACA...AGACCCCCTGTCTCAGTCACCCAGATCCCAGCTCAGGTCAGCCCAACTGTGTC   539
SCPP9-CI       CGCTCCTTCCTCAGAAGGAACACTGCGAGACCACCTGTCTCAATCACCCAGATGCCAGCTCAGCTCAGCCCTACTGTGTC   533
SCPP9-C1˙CC    CGCTCCTTCCTCAGAAGGACCACAGCAAGACCACCTGTTAATAATTATTTTATGCCAGCTCAAGTCAACCCTGCTGTGTC   524
SCPP9-C2˙CC    CGCTCTTTTCTCAGAAGGACCACTGCAAGACCACCTGTCTTAATCACCCAGATGCCAGCTCAGGTCAACCCTGCTGTGTC   530
SCPP9-C1˙SG    CGCTCCTTCCTCAGAAGGACCACAGCAAGA...CCTGTCTCAATCACCCAGATGCCAGTTCAGATCAACCCTGCTGTGCC   521
SCPP9-C2˙SG    CGCTCCTTCCTCAGAAGGACCACTGCAAGACCACCTGTCTCAATCACCCAGATGCCAGCCCAGGTCAACCCTGCTGTGTC   539
SCPP9-C1˙SA    CGCTCCTTCCTCAGAAGGACCACAGCAAGA...CCTGTCTCAATCACCCAGATGCCAGCTCAGATCAACCCTGCTGTGCC   521
SCPP9-C2˙SA    CGCTCCTTCCTCAGAAGGACCACTGCAAGACCACCTGTCTCAATCACCCAGATGCCAGCCCAGGTCAACCCTGCAGTGTC   449
SCPP9-C1˙SR    CGCTCCTTCCTCAGAAGGACCACAGCAAGA...CCTGTCTCAATCACACAGATGCCAGCTCAGATCAACCCTGCTGTGCC   521
SCPP9-C2˙SR    CGCTCCTTCCTCAGAAGGACCACTGCAAGACCACCTGTCTCAATCACCCAGATGCCAGCCCAGGTCAACCCTGCTGTGTC   533

conservation   !!!!!!!************
SCPP9˙DR       TGGAAATACTGTTGGCTAA   552
SCPP9˙LW       TGGAAAT............   552
SCPP9˙PP       TGGAAAT............   546
SCPP9-CI       TGGAAATTCTGTGGGGTAA   552
SCPP9-C1˙CC    TGGAAACCCTGTTGGGTAA   543
SCPP9-C2˙CC    TGGAAATCCTGTTGGGTAA   549
SCPP9-C1˙SG    TGGAAATCCTGTTGGGTAA   540
SCPP9-C2˙SG    TGGAAATCCTGTTGGGTAA   558
SCPP9-C1˙SA    TGGAAATCCTGTTGGGTAA   540
SCPP9-C2˙SA    TGGAAATCCTGTTGGGTAA   468
SCPP9-C1˙SR    TGGAAATCCTGTTGGGTAA   540
SCPP9-C2˙SR    TGGAAATCCTGTTGGGTAA   552
```

X non conserved

X ≥55% conserved

X ≥85% conserved
