# Supplementary material for: A Genomic Survey of SCPP Family Genes in Fishes Provides Novel Insights into the Evolution of Fish Scales
Source: Int J Mol Sci. 2017 Nov 16;18(11):2432. doi: 10.3390/ijms18112432 (PMC5713400; doi:10.3390/ijms18112432)
Supplement: Supplementary file 1 [file ijms-18-02432-s001.zip › ijms-236743-supplementary/Figure S4.SCPP7.cds.pdf]

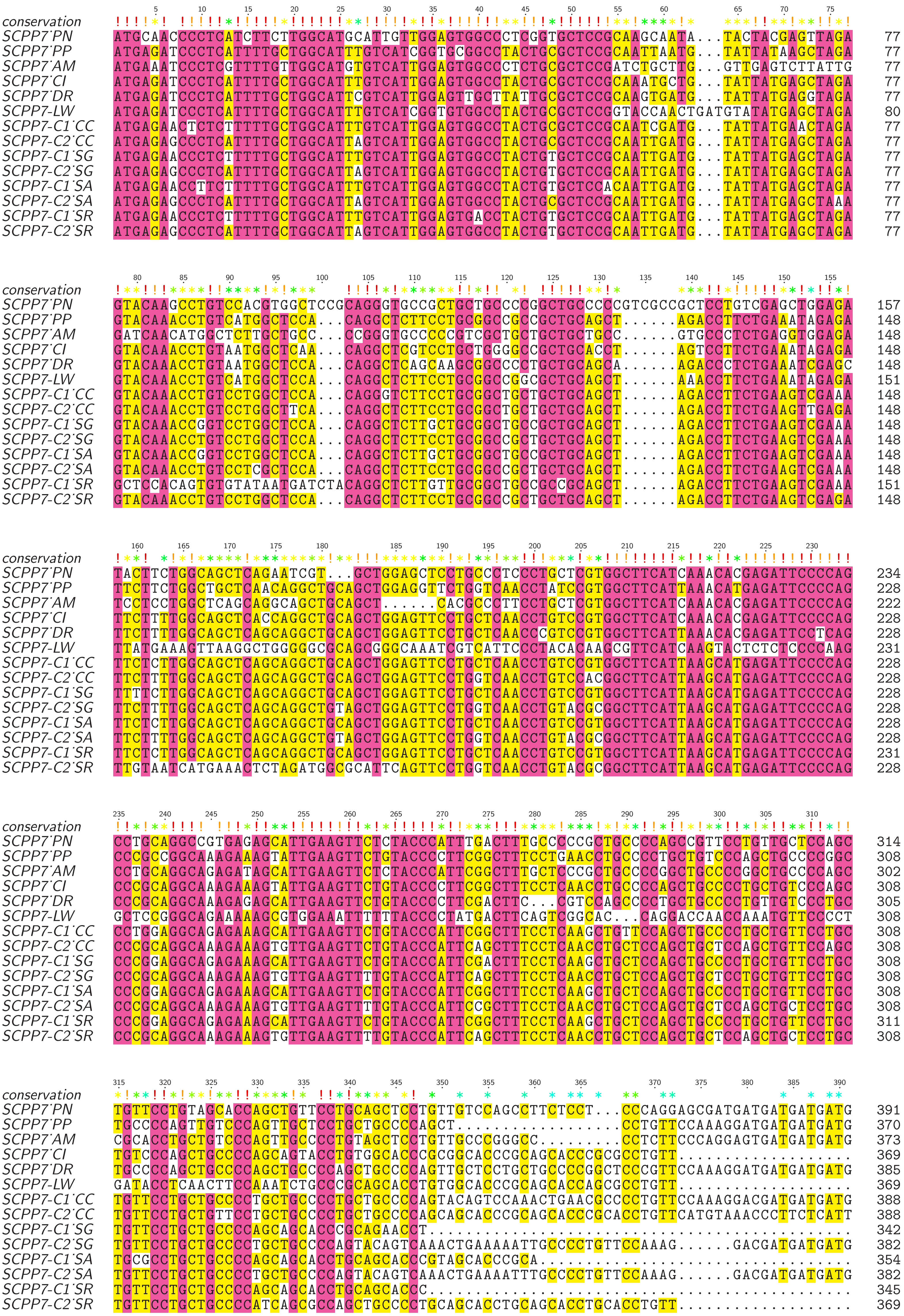
conservation
SCPP7_PN
SCPP7_PP
SCPP7_AM
SCPP7_CI
SCPP7_DR
SCPP7-LW
SCPP7-C1_CC
SCPP7-C2_CC
SCPP7-C1_SG
SCPP7-C2_SG
SCPP7-C1_SA
SCPP7-C2_SA
SCPP7-C1_SR
SCPP7-C2_SR

```
                   395      400     405
conservation          *       **
SCPP7˙PN         ACCATGATGACTAA   405
SCPP7˙PP         ATCATGATGACTAA   384
SCPP7˙AM         ATGAGAACGAT...   384
SCPP7˙CI         ..............   369
SCPP7˙DR         ATCAAGATGACTAA   399
SCPP7-LW         ..............   369
SCPP7-C1˙CC      ATCATGATGACTAA   402
SCPP7-C2˙CC      CAAAATCAGAC...   399
SCPP7-C1˙SG      ..............   342
SCPP7-C2˙SG      ATCATGATGACTAA   396
SCPP7-C1˙SA      ..............   354
SCPP7-C2˙SA      ATCATGATGACTAA   396
SCPP7-C1˙SR      ..............   345
SCPP7-C2˙SR      ..............   369
```

X non conserved

X $\geq$55% conserved

X $\geq$85% conserved
