# Supplementary material for: A Genomic Survey of SCPP Family Genes in Fishes Provides Novel Insights into the Evolution of Fish Scales
Source: Int J Mol Sci. 2017 Nov 16;18(11):2432. doi: 10.3390/ijms18112432 (PMC5713400; doi:10.3390/ijms18112432)
Supplement: Supplementary file 1 [file ijms-18-02432-s001.zip › ijms-236743-supplementary/Figure S6.SCPP6.cds.pdf]

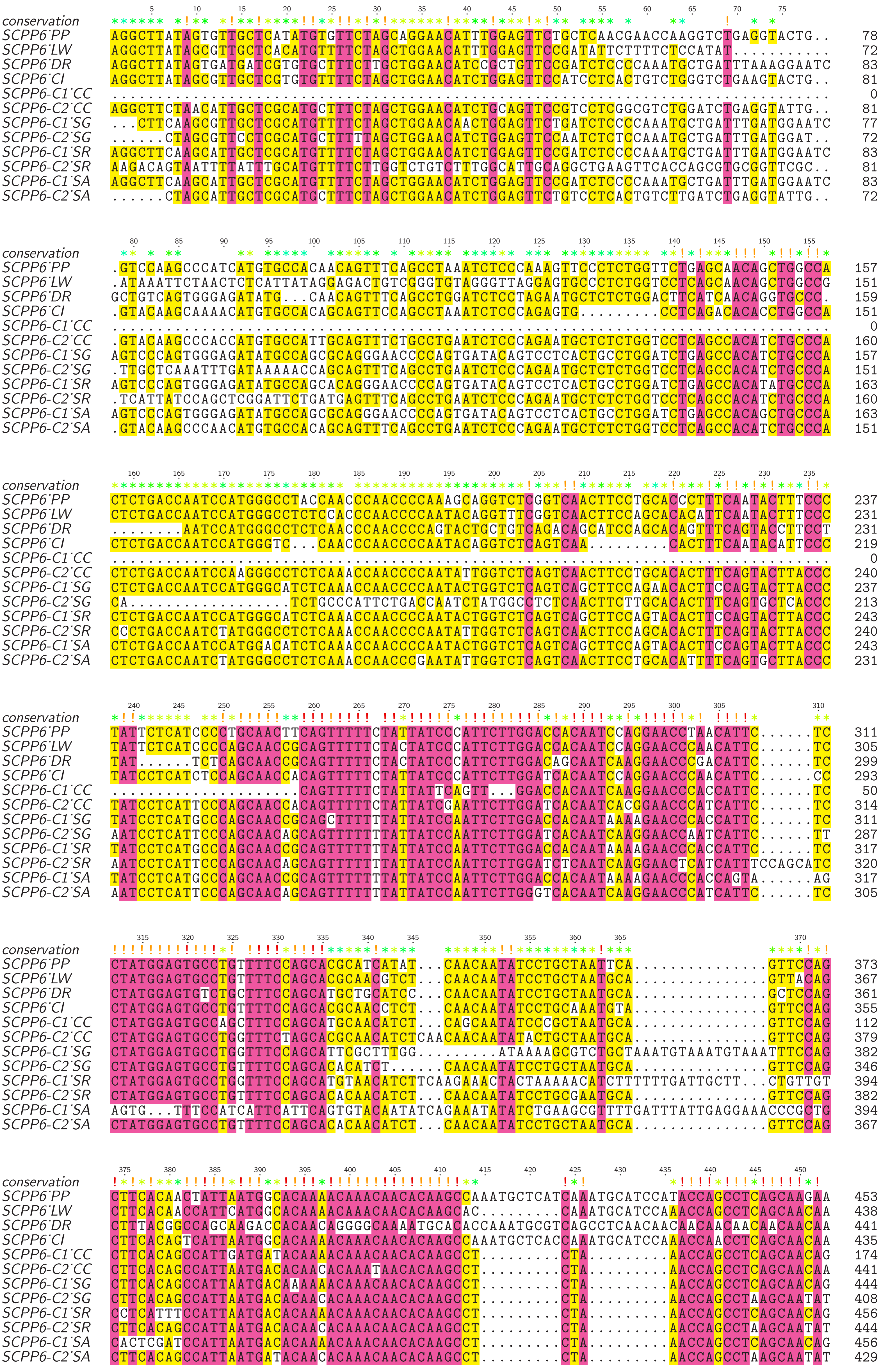
conservation
SCPP6 PP
SCPP6 LW
SCPP6 DR
SCPP6 CI
SCPP6-C1 CC
SCPP6-C2 CC
SCPP6-C1 SG
SCPP6-C2 SG
SCPP6-C1 SR
SCPP6-C2 SR
SCPP6-C1 SA
SCPP6-C2 SA

```
                455  460  465                   470  475  480  485  490  495  500  505  510  515  520
conservation    !!****** ***            ******************** ******** ************ *************
SCPP6˙PP        CAGGTTCAACAA............TTTCTTTACATGGTTCCACAGATTCAACAACGAATGGCTGGACCATATGGTGGATT   521
SCPP6˙LW        CAGGTTCAACAA............TTTCTTTACATGGTTCCACAGACTCAACAACGAATGGCTGGACCATATGGTGGATT   506
SCPP6˙DR        CAGTTTCAACCC............ATTTTTTATATGGTTCCTCAAATGCCACAGCGAATGGCTGGATCATATGGTGGACT   509
SCPP6˙CI        CAGGTTCAACAA............TTTCTTTACATGGTTCCACAGATTCAACAGCGAATGGCTGGACCATATGGCGGATT   503
SCPP6-C1˙CC     CAGGTTCATCAA............TTTCTTTACATGGTTCCACAGATTCAACAACGAATGGCTGGACCATATGGTGGATT   242
SCPP6-C2˙CC     CAGGTATCAACACAAACATTAATATTATTATATTATATT.........................................   480
SCPP6-C1˙SG     CAGGTTCATCAA............TTTCTTTACATGGTTCCACAAATTCAACAACGAATGGCTGGACCATATGGTGGATT   512
SCPP6-C2˙SG     CAATCTTAAACA....................................................................   420
SCPP6-C1˙SR     CAGGTTCATCAA............TTTCTTTACATGGTTCCACAAATTCAACAACGAATGGCTGGACCATATGGTGGATT   524
SCPP6-C2˙SR     CAACCAAGTCAG....................................................................   456
SCPP6-C1˙SA     CAGGTTCATCAA............TTTCTTTACATGGTTCCACAAATTCAACAACGAATGGCTGGAGCATATGGTGGATT   524
SCPP6-C2˙SA     CAACCAAGTCAG....................................................................   441

                   525  530  535  540  545  550  555  560  565  570  575  580  585  590  595  600
conservation    * *** ******* *****                    ** *       *  *   *    * *      *
SCPP6˙PP        GAGCTCTGAGGAACTTCAGAACATGGGACAAATGGGTCCTGTTGACATGCACATGCCTGCGTTTAGGGGCAATGTGTTGC   601
SCPP6˙LW        GAGCTCTGAGGAA...................................................................   519
SCPP6˙DR        GAGCTCTGAGGAACTTCAGAACATGGGC.........CGTGTTAACATGCACCTACCTGCGATTAGAGGAAATGTGTTTC   580
SCPP6˙CI        GAGCTCCGAGGAAATTCAGAACATGGGACATATGGGCCATGTTAACATGCACATGCCTGAGTTTAGAGGCAATGTGTTTC   583
SCPP6-C1˙CC     GCGCTCTGAGGAACTTCAGTGACCTAAAAAAGAAGACTTTGATATATTTGATATATATCTCATAACTTCTGACACCACTG   322
SCPP6-C2˙CC     ................................................................................   480
SCPP6-C1˙SG     GCGCTATGAGGAACTTCAGAACATGGGACGCATGCGTCGTGTTGACTTGCACATGCCTGCACTTAGAGGCGATGTTTGTC   592
SCPP6-C2˙SG     ................................................................................   420
SCPP6-C1˙SR     GAGCTCTGAGGAACTTCAGAACATGGGACGCATGCGTCGTGTTGACTAGCACATGCCTGCACTTAGAGGCAATGTTTTTC   604
SCPP6-C2˙SR     ................................................................................   456
SCPP6-C1˙SA     GCGCTATGAGGAACTTCAGAACATGGGATACATGCGTCGTGTTGACTTGCACATGCCTGCACTTAGAGGCAATGTTTTTC   604
SCPP6-C2˙SA     ................................................................................   441

                   605  610  615  620  625  630  635  640  645  650  655  660  665  670  675  680
conservation                   *      * *     *             *                      *
SCPP6˙PP        CTGTAGCTGGACCACAACCTGTTGTACCTGTTAACCCTTTAAGACCAGCCAATACCTTCCACATCAACACCTACCCTGCC   681
SCPP6˙LW        ................................................................................   519
SCPP6˙DR        CTGTATCTGGACCTCAAACTGTTGTACCCATTGGGCCAGTAAGACCG...............CCCAACCCCTTCCCTGCC   645
SCPP6˙CI        CTGTAGCTGGACCACAACCTGTTGTACCTGTTAGCTCTTTAGGACCAGCCAATACCTTCCACATCAACACCTTCCCTGCC   663
SCPP6-C1˙CC     TATCTTTATTTTTTAATGTATGTATTGGAATGAATAAACCTTAGCCTAAATCTCTAATTAAGACACACAAA.........   393
SCPP6-C2˙CC     ................................................................................   480
SCPP6-C1˙SG     CTGTAGCTGGACCACAACCTGTTGTACCTGTTAGCCCTTTAAGACTGACCAAGACCTTCCACCCCAGCACCTTT......   666
SCPP6-C2˙SG     ................................................................................   420
SCPP6-C1˙SR     CTGTAGCTGGACCACAACCTGTTGTACCTGTTAGCCCTTTAAGACCGACCAAGACCTTCCACCCCAGCACCTTT......   678
SCPP6-C2˙SR     ................................................................................   456
SCPP6-C1˙SA     CTGTAGCTGGACCACAACCTGTTGTACCTGTTAGCCCTTTAAGACCAACCAAGACCTTCCACCCCAGCACCTTT......   678
SCPP6-C2˙SA     ................................................................................   441

                   685  690  695  700  705  710  715  720  725  730  735  740  745  750  755  760
conservation                             *             *       *   **     * *        ***
SCPP6˙PP        ACTGGAATGACATTCTCGACCAATACTGAGGCCCAGCCACCTAGCATTGTAGCTGTACCACCCAGAGGGGATGCCATACT   761
SCPP6˙LW        ................................................................................   519
SCPP6˙DR        ACTGGAATGACCTACCCGGGCATTACTGAGCTCCAGCAACCCAGCATTATAGCCGTGCCTCCTAGCAGAGATGCCATACC   725
SCPP6˙CI        AATGGAATGACCTTCTCGGGCACTACTGAGGTTCAGCCACCAAGCGTTGTAGCTGTACCACCCAGTTGGGATGCCATACC   743
SCPP6-C1˙CC     ........GAACATGAGACGTCTTCTGAAGTTGAGCTACCCAGCGTTCTAGCTGTACCACCCAGTGGGGATGCCATATC   464
SCPP6-C2˙CC     ................................................................................   480
SCPP6-C1˙SG     ............CTGACCATTCTGACCATTTTTCAGCTACCCAGCATTGCAGCTGCACCACCCAGTGGGGATGCCATATC   734
SCPP6-C2˙SG     ................................................................................   420
SCPP6-C1˙SR     ............CTGACCATTCTGACCATTTGTGCTACCCGCTCAGATTGTGCTAACTCCCATCAAAAAGAT........   738
SCPP6-C2˙SR     ................................................................................   456
SCPP6-C1˙SA     ............TTGACCATTCTGACGATTTTTCAGCTACCCAGCGTTGTAGCTGTACCACCCAGTGGGGATGCCATATC   746
SCPP6-C2˙SA     ................................................................................   441

                   765  770  775  780  785  790  795  800  805  810  815  820  825  830  835  840
conservation        *  *    ***  **    *            *       **     *
SCPP6˙PP        TGCTACTGGTGGATCCCATCCAAATAATGGAGTGCTGCCTAGTAGGAATGCTATCAAGCAGGACCCTGAGCGAGTGCCGT   841
SCPP6˙LW        ................................................................................   519
SCPP6˙DR        TGCTACTGGTGGATCCCATCCAAATAGTGCAGTGCTGCCTAGTAGGATTTCTGGCACTCAGGATCGTGATCGAGCACCAT   805
SCPP6˙CI        TGCTACTGGTGGATCCCATCCAAATAATGGAGCGCAGCCTAATAGGATTGCTACTGTGCAGGACTATGAGCGAGTGCCTT   823
SCPP6-C1˙CC     TGTGACTGGTGGATCCAATCCAAATAATAGAGCACCGCCTAGTAGGACTGCTAACATGCAGGACCATGAGTGAGTGCCGT   544
SCPP6-C2˙CC     ................................................................................   480
SCPP6-C1˙SG     TGCGACTGGTGGATCCCATCCAAATAATGGAGCACTGCCAAGTAGGATTGCTAGCGTACAGGACCGTGAGCAAGTGCCGT   814
SCPP6-C2˙SG     ................................................................................   420
SCPP6-C1˙SR     ....ATAGAACAATCTGATAGGTAGCATGCTTCGTTGGTACACCGGCAACTTCTTAAGTTCAGCTACCCAGCGTTGTAGC   814
SCPP6-C2˙SR     ................................................................................   456
SCPP6-C1˙SA     TGCGACTGGTGTATCCCATCCAAATAACGGAGCACTGCCTAGTAGGATTGGTTAC.........CGTGAGCAAGTGCCGT   817
SCPP6-C2˙SA     ................................................................................   441

conservation
SCPP6˙PP        GT   843
SCPP6˙LW        ..   519
SCPP6˙DR        GT   807
SCPP6˙CI        GT   825
SCPP6-C1˙CC     GT   546
SCPP6-C2˙CC     ..   480
SCPP6-C1˙SG     GT   816
SCPP6-C2˙SG     ..   420
SCPP6-C1˙SR     TG   816
SCPP6-C2˙SR     ..   456
SCPP6-C1˙SA     GT   819
SCPP6-C2˙SA     ..   441
```

X non conserved

X $\geq$55% conserved

X $\geq$85% conserved
