# Supplementary material for: A Genomic Survey of SCPP Family Genes in Fishes Provides Novel Insights into the Evolution of Fish Scales
Source: Int J Mol Sci. 2017 Nov 16;18(11):2432. doi: 10.3390/ijms18112432 (PMC5713400; doi:10.3390/ijms18112432)
Supplement: Supplementary file 1 [file ijms-18-02432-s001.zip › ijms-236743-supplementary/Figure S7.SCPP1.cds.pdf]

```
SCPP1_CI      ATGAAACCTGTCATCCTGATTCTCTGTCTGCTGGGAGCAGCCTATGCTAATCCAATCTTGCATAAAATGGCTATGGAGAT   80
SCPP1_AM      ................................................................................    0
SCPP1_DR      ATGAAGTCTGCCCTATTGATTCTTTGTCTGTTGGGAGCAGCCTGTGCTAATCCAATCTTGCATAAAGTGGCTATGGAGAT   80
SCPP1_IP      ATGAAGCTGGCTTTCGTGATTCTCTGCCTGCTCGGAGCTGCCGGTGCAAATCCAATTTTGCAC.........ACGGATAT   71
SCPP1_LW      ATGAAACCTGCCATACTGATTCTATGTCTGCTGGGAACAGCCTATGCTAATCCAATCTTGCATAAAATGGCTATGGAGAT   80
SCPP1_PN      ATGAAGTTTGCCATTGTAATTCTCTGCCTGCTGGGAACAACCAGTGCTAATCCCATCTTATACAAAGTGACCACTGAGAT   80
SCPP1_PP      ATGAAACCTGCCATACTGATTCTATGTCTGCTGGGAGCAGCCTACGCTAATCCAATCTTGCATAAAATGGCTATGGAGAT   80
SCPP1-C1_CC   ATGAAACCTGCCATGTTGATTCTCTGTCTGCTGGGAGCAGCCTATGCTAATCCAATCTTGCATAAAATGGCTATGGAGAT   80
SCPP1-C2_CC   ATGAAACATGCCATATTGATTCTCTGTCTGCTGGGAGCAGCCTATGCTAATCCAATCTTGCATAAAACGGCTATGGAGAT   80
SCPP1-C1_SR   ................................................................................    0
SCPP1-C2_SR   ATGAAACCTGCCATATTGATTCTCTGTCTGCTGGGAGCAGCCTATGCTAATCCAATCTTGCATAAAAAGACTATGGAGAT   80
SCPP1-C1_SA   ATGAACCCTGCCATATTGATTCTCTGTCTGCTGGGAGCAGCCTATGCTAATCCAATCTTGCATAAAATGGCTATGGAGGT   80
SCPP1-C2_SA   ATGAAACCTGCCATACTGATTCTCTGTCTGCTGGGAGCAGCCTATGCTAATCCAATCTTGCATAAAAAGACTATGGAGAT   80
SCPP1-C1_SG   ATGAAACCTGCCATATTGATTCTCTGTCTGCTGGGAGCAGCCTATGCTAATCCAATCTTGCATAAAATGGCTATGGAGGT   80
SCPP1-C2_SG   ATGAAACCTGCCATATTGATTCTCTGTCTGCTGGGAGCAGCCTGTGCTAATCCAATCTTGCATAAAAAGACTATGGAGAT   80

SCPP1_CI      GATTCAACATGCATCCAACTCTTCAGAAAGCTCTTCAATATCTGAATCCTCCGACCAGAGTAACACCTCAGAACCTTCAG  160
SCPP1_AM      ................................................................................    0
SCPP1_DR      GATTCAACATGCATCTAACTCTTCGGAAAGCTCCTCAATATCTGAATCCTCAGACCAGAGTAATACCTCAGAACCTTCGG  160
SCPP1_IP      GATGGAGACCGCATCGAACTCTTCACAGACCTCCTCGATGTCTGCATCTACTGAAGAA......ACCGTTGCCATAGACC  145
SCPP1_LW      GATTCAACATGCATCCAACTCTTCGGAAAGCTCTTCAATATCTGAATCCTCCGACCAGAGTAATACCTCAGAACCTTCAG  160
SCPP1_PN      GATGGAACATGCATCCAACTCT......ACCTCATCAGTGTCTGAATCCTCTGAAGAAAGCGATACATCAGAA......C  148
SCPP1_PP      GATTCAACATGCATCCAACTCTTCGGAAAGCTCTTCAATATCTGAATCCTCCGACCAGAGTAATACCTCAGAACCTTCAG  160
SCPP1-C1_CC   GATTCAACATGCATCCAACTCTTCGGAGAGCTCTTCAATATCTGAATCCTCTGACCAGAGTAATACCTCAGAACCTTCAG  160
SCPP1-C2_CC   TATTCAACATGCATCCAACTCTTCGGAAAGCTCTTCGATGTCTGAATCTTCCGACCAGAGTAATACCTCAGAACTTTCAG  160
SCPP1-C1_SR   ..........................................................CAGGATTCATCCCCACCAAGCG   22
SCPP1-C2_SR   GATTCAACATGCATCCAACTCTTCGGAAAGCTCTTCAATGTCTGAATCCTCCGACCAGAGTAATACCTCAGAACTTTCAG  160
SCPP1-C1_SA   GATTCAACATGCATCCAACTCTTCGGAAAGCTCTTCAATATCTGAATCCTCCGACCAGAGTAATATCTCAGAACCTTCAG  160
SCPP1-C2_SA   GATTCAACATGCATCCAACTCTTCGGAAAGCTCTTCAATGTCTGAATCCTCCGACCAGAGTAATACCTCAGAACTTTCAG  160
SCPP1-C1_SG   GATTCAACATGCATCCAACTCTTCGGAAAGCTCTTCAATATCTGAATCCTCCGACCAGAGTAATACCTCAGAACCTTCAG  160
SCPP1-C2_SG   GATTCAACATGCATCCAGCTCTTCGGAAAGCTCTTCAATGTCTGAATCCTCCGACCAGAGTAATACCTCAGAACTTTCAG  160

SCPP1_CI      AAGAGAAGTCCAAGGAAAATGCCTCCGACAGCAGTAGCTCAGAATCGCTTGAATCTGACTCAGATGAACCGATCTCT...  237
SCPP1_AM      ................................................................................    0
SCPP1_DR      AAGAGAAGTCAGAGGAAAATGTTTCCGACAGCAATAGCTCTGAATCACTTGAATCTGAATCAGATGAACCGATATCTAAA  240
SCPP1_IP      AAGATAGCTCTCAGGAAAATACTTCAGAGGACACCACCTCAGAATCCATGGAATCGAATTCATCTGAGAAGACCTTGGAA  225
SCPP1_LW      AAGAGAAGTCCAAGGAAAATGTCTCAGACAGCAATAGCTCAGAATCGCTTGAATCTGACTCGGATGAAACGATCTCTAAA  240
SCPP1_PN      ACAACAGCTCACCGGAAAATACTTCTGAGAACAATACGTCAGAATCGCTGGAGTCTGCATCCGATGATCAGACTTCAGAC  228
SCPP1_PP      AAGAGGTAAGC.....................................................................  171
SCPP1-C1_CC   ATGAGAAATCTAAGGAAACTGTCTCCGACAGCAGCAGCTCAGAATCAACTGAATCAGACTCTAATGAACCGATCTCA...  237
SCPP1-C2_CC   AAGAGAAGTCTACGGAAACTGTCTCTGACAGCAATAGTTCAGAATCAATTGAATCGGCCTCAAATGAACCGATTTCT...  237
SCPP1-C1_SR   AGACTAAGTCTAAGGAAACTGTCTCCGACAGCAATAGCTCAGAATCAACTGAATCAGACTCTAATGAACCGATCTCT...   99
SCPP1-C2_SR   AAGAGAACTCTAAGGAAACTGTCTCCGACAGCAATAGTTCAGAATCAATTGAATCGGACTCAAATGAACCGATTTCA...  237
SCPP1-C1_SA   ATGAGAAGTCTAAGGAAACTGTCTCCGACAGCAATAGCTCAGAATCAACTGAATCAGACTCTAATGAACCGATCTCT...  237
SCPP1-C2_SA   AAGAGAAGTCTAAGGAAACTGTCTCCGACAGCAATAGTTCAGAATCAATTGAATATGACTCAAATGAACCGATTTCA...  237
SCPP1-C1_SG   ATGAGAAGTCTAAGGAAACTGTCTCCGACAGCAATAGCTCAGAATCAACTGAATCAGACTCTAATGAACCGATCTCT...  237
SCPP1-C2_SG   AAGAGAAGTCTAAGGAAACTGTCTCAGAGAGCAGTAGTTCAGAATCAATTGAATCAGACTCAAATGAACCGATTTCA...  237

SCPP1_CI      ...AGCGAAAGCCATTCTGTGGAAAGACTGATTGGAAAAAGCGACACTTCACTGACATCAGATAACACTCAAAGCAGTAA  314
SCPP1_AM      .........TCAGATTCCATGTTGGTTCAGTTTGGTACAGGTGAGCCTGGAATGACCACAGACAACAGCCAAGGAAGCCA   71
SCPP1_DR      GAGAGTGAAAGCCATTCTGTGGAAAGTCTGATTGGAAAAAGCGAGACCGCACTGACAGCCGACAACACTCAAAGCAGTAA  320
SCPP1_IP      ACAAGCCAAAGCAACTCGCTGGAAGAACGGTTTGGCAATGGAGAGGCTGGAATGACCGTCGATAACAGCCAAGGAAGCAC  305
SCPP1_LW      GAGAGCGAAAGCCATTCTGTGGAAAATTTGATTGGAAAAAGCGAGGCTGCACTGACATCGGATAACACTCAAAGCAGTAA  320
SCPP1_PN      ATGAGCCGGAGTCACTCCCTGGAAGCTCGGTTTGGCACAGGTGAGACAGGAATGACCACAGATGACAGCCAAGGCAGCCT  308
SCPP1_PP      ................................................................................  171
SCPP1-C1_CC   ...AGTGAAAGCCATTCTGTGGAAAGTCTGATTGGAAAAAGTGAGACTGCACTGACATCAGATAACACTCAAAGCAGTAA  314
SCPP1-C2_CC   ...AGTGAAAGCCATTCAGTGGAAAGTCTGTCTGGAAAAAGCGAGACTGCACTGACATCAGACAACACTCAAAGCAGTAA  314
SCPP1-C1_SR   ...AGTGAAAGCCATTCTGTGGAAAGTCTGATTGGAAAAAGTGAGACTGCACTGACATCAGATAACACTCAAAGCAGTAA  176
SCPP1-C2_SR   ...AGTGAAAGCCATTCTGTGGAAAGTCTGACTGGAAAAAGCGAGACTGCACTGACATCAGATAACACTCAAAGCAGTAA  314
SCPP1-C1_SA   ...AGTGAAAGCCATTCTGTGGAAAGTCTGATTGGAAAAAGTGAGACTGCACTGACATCAGATAACACTCAAAGCAGTAA  314
SCPP1-C2_SA   ...AGTGAAAGCCATTCTGTCGAAAGTCTGACTGGAAAAAGCGAGACTGCACTGACATCAGATAACACTCAAAGCAGTAA  314
SCPP1-C1_SG   ...AGTGAAAGCCATTCTGTGGAAAGTCTGATTGGAAAAAGTGAGACTGCACTTACATCAGATAACACTCAAAGCAGTAA  314
SCPP1-C2_SG   ...AGTGAAAGCCATTCTGTGGAAAGTCTGACTGGAAAAAGCGAGACTGCACTGACATCAGATAACACTCAAAGCAGTAA  314

SCPP1_CI      AGAGAACGTTCGTAGGGGCTGGATCTACACCCTCAAA............TGGGTCCATATGAACAATGGTGGCAAAGTGC  382
SCPP1_AM      GGAGAACATGCGCAAGAACTGGGTTCATCTCATCAAC............GTCAAAATGGCCAGTAAAGAGGACACGGAGG  139
SCPP1_DR      AGAGAACATCCGTAGGGGCTGGATCTACACCCTCAAA............TGGGTCCAACCAAACAACAAC...ATTGTGC  385
SCPP1_IP      AGAGATCATGCGCAAGAGCCCAGAGAAAAACTCCAAG...............AGCATCAGCAGCAGTAGTGAGAGCAGTG  370
SCPP1_LW      AGAGAATGTCCGTAGGGGCTGGATCTACACCCTCAAA............TGGGTCCATACGAATAACAGCGGCAAAGTGC  388
SCPP1_PN      GGAGAACATGCGCAAGCTCCACCCATTCATACTGAGGTTCTAATGAAGCTGGATTCATCTCATCAACGTCAAAGTGCTCA  388
SCPP1_PP      ................................................................................  171
SCPP1-C1_CC   CGAGAACGTTCGTAAGGGCTGGATCTACACCCTCAAA............TGGGTTCATAAGAACAACAATGCCATTGTGC  382
SCPP1-C2_CC   AGAGAATGTTCGTAGGGGCTGGATCTACACCCTCAAA............TGGGTCCATATTAACAACAACGGCAAAGTGC  382
SCPP1-C1_SR   AGAGAACGTTCGTAGGGGCTGGATTTACACCCTCAAA............TGGGTCCATAAGAACAACAATTCCATTGTGC  244
SCPP1-C2_SR   AGAGAATGTTCGTAGGGGCTGGATCTACACCCTCAAA............TGGGTCCATATTAACAACAACGGCAAAGTGC  382
SCPP1-C1_SA   AGAGAACGTTCGTAGGGGCTGGATTTACACCCTCAAA............TGGGTCCATAAGAACAACAATGCCATTGTGC  382
SCPP1-C2_SA   AGAGAATGTTCGTAGGGGCTGGATCTACACCCTCAAA............TGGGTCCATATTAACAACAACGGCAAAGTGC  382
SCPP1-C1_SG   AGAGAACGTTCGTAGGGGCTGGATTTACACCCTCAAA............TGGGTCCATAAGAACAACAATGCCATTGTGC  382
SCPP1-C2_SG   AGAGAATGTTCGTAGGGGCTGGATCTACACCCTCAAA............TGGGTCCATATTAACAACAACGGCAAAGTGC  382
```

```
               385  390  395  400  405  410  415  420  425  430  435  440  445  450  455  460
conservation  ******************* ***********  * ***** ** **        *       ** *********** **
SCPP1˙CI      AAGCGACCAGCCAGCCAGACGAGAATTACATCAAATCCACCAGTGATGCATCAAAAAAAAGTGAAAGCAGTGAATCGAGT 462
SCPP1˙AM      AAGTGACCGACCAACCAGACGAGGAGGACAAAGACACGACAGAGGAC...............CAGACCTCCGAGAGCAGC 204
SCPP1˙DR      AACCAACCGGCCAGCCACATGAAAATGACATCCAGTCCACTAGTGACGCATCCCAAATAAGTGAGAGCAGCGAATCAAGC 465
SCPP1˙IP      AGTCTACTGAGGGTCAAGGCAACAATTCAACAAGCAGCAGCAGCGAGAGCAGTGAGAGCAGCGAAAGCAGTGAGAGCAGC 450
SCPP1˙LW      AAGCGACCAGCCAGCCGGAC..............................ATCAAATCCAGTGAAAGCAGTGAATCAAGT 438
SCPP1˙PN      GCGAGGAGGACAAAGACACGACAGAGGACCTCTCCTCTAAC........................AGCAGTGAATCTACA 444
SCPP1˙PP      ................................................................................ 171
SCPP1-C1˙CC   AAGCGACCAGCCAGCCAGACGAGAATGACATCAAATCCACTAGTGACGCATCAAAAAAAAGTGAAAGCAGTGTTTCAAGT 462
SCPP1-C2˙CC   AAGCGACCAGCCAGCCAGATGAGAATGACATCAAGTCCACTAGTGATGCGTCAAAAAAAAGTGAAAGCAGTGAATCAAGT 462
SCPP1-C1˙SR   AAGCGACCAGCCAGCCAGAAGAGAATGACATAAAATCCACTAGTGACGAATCAAAAAAAAGTGAAAGCAGTGAATCAAGT 324
SCPP1-C2˙SR   AAGCGACCAGCCAGCCAGAAGAGAATGACATCAAATCCACTAGTGACGCGTCAAAAAAAAGTGAAAGCAGTGAATCAAGT 462
SCPP1-C1˙SA   AAGCGACCAGCCAGCCAGAAGAGAATGACATCAAATCCACTAGTGACGAATCAAAAAAAAGTGAAAGCAGTGAATCAAGT 462
SCPP1-C2˙SA   AAGCGACCAGCCAGCCAGAAGAGAATGACATCAAATCCACTAGTGACGCATCAAAAAAA......AGCAGTGAATCAAGT 456
SCPP1-C1˙SG   AAGCGACCAGCCAGCCAGAAGAGAATGACATCAAATCCACTAGT..................GAAAGCAGTGAATCAAGT 444
SCPP1-C2˙SG   AAGTGACCAGCCAGCCAGAAGAGAATGAC................................................... 411

               465  470  475  480  485  490  495  500  505  510  515  520  525  530  535  540
conservation  ****** ****** ********* * *** *** * *************  ***** *********************
SCPP1˙CI      GAAAGCCAGGAAACGGTTGTTCTGAACACAAAGGAAGAGGATAACAGTGCCGACACGAGTGAAAGCACTGAACACAGT.. 540
SCPP1˙AM      GAGTCCACGGAGAAGCCCACCCCCAGCAGCAGCAGCAGCAGCAGCACAGAGGACAGC...............AGGGCGGT 269
SCPP1˙DR      GAAAGCCAGGAAAAGGTTGTCGTGAACAAGATGCAAGTGGATAACAGTGACGACACCAGTGAAAGCGCTGAACACAATGG 545
SCPP1˙IP      GAGTCCACTGAAAACCCGGAGAAGAACTCCAACAGCAGCAGCAACAGCAGCAGTGAGAGCAAAAGCGTCGAGAACAGATC 530
SCPP1˙LW      GAAAGCCAGGAAACAGTTGTTCTGAGCACAAAGGAAGAGAATAACGGTGCCGACACAAGTGAAAGC.............. 504
SCPP1˙PN      GAGAGTCAGGAGCAGCAAACCCACAGCAGCAGCAGCAGCAGCAGCACAGAGTCCAGC...AGCAAAGACAGCAGGGC 521
SCPP1˙PP      ................................................................................ 171
SCPP1-C1˙CC   GAAAGCCAGGAAATGGCTGTTCTAAACACAAAGCAAGAGGATAACAGTGTGGACACAAGTGAAAGCACTGAAAACAGTTA 542
SCPP1-C2˙CC   GAAAGCAAGGAAACAGTTGTTCTGAACACCAAGGAGGAGGATAACAGTGCCGATACAAGTGAAAGCACTGAAAACAGTTA 542
SCPP1-C1˙SR   GAAAGCCAGGAAATGGTTGTTCTAAGCACAAAGGAAGAGGATAAGAGTGCGGACATAAGTGAAAGCACTGAAAACAGTTA 404
SCPP1-C2˙SR   GAAAGCAAGGAAATGGTTGTTCTGAACACAAAAAAGGAGGATAACAGTGCCGACACAAGTGAAAGCACTGAAAACAGTTA 542
SCPP1-C1˙SA   GAAAGCCAGGAAATGGTTGTTCTAAGCACAAAGGAAGAGGATAAGAGTGTGGACACAAGTGAAAGCACTGAAAACAGTTA 542
SCPP1-C2˙SA   GAAAGCAAGGAAATGGTTGTTCTGAACACAAAGGAGGAGGATAACAGTGCCGACACAAGTGAAAGCACTGAAAACAGTTA 536
SCPP1-C1˙SG   GAAAGCCAGGAAATGGTTGTTCTAAGCACAAAGGAAGAGGATAAGAGTGCGGACACAAGTGAAAGCACTGAAAACAGTTA 524
SCPP1-C2˙SG   ...AGCAAGGAAATGGTTGTTCTGAACACAAAGGAGGAGGATAACAGTGCCGACACAAATGAAAGCACTGAAAACAGTTA 488

                     545  550  555  560  565  570           575  580  585  590  595  600  605
conservation  *   *********************** *****          ***************** ************** ****
SCPP1˙CI      ....GTTGAAGGTACTGAGTATAATACGAGCAAC.........AGCAGCAGCAGCAGCAGTAGCAGTGAAAGCACCGAGT 607
SCPP1˙AM      GGTGGTGGACAGCTCTGAGAACAGCCACAGCAAC............AGCAGCAGCAGCAGCAGCAGCGAGAGCCACGAGT 337
SCPP1˙DR      TGTTGTT...GCTACTGAATACAGCAACAGTAAC.........AGCAGCAGCAGCAGTAGCAGCAGTGAAAGCACCGAGT 613
SCPP1˙IP      AACGATCGACAGCGATGAGAACAGCGCCCTGAAA...AGCAGCAACAGCTCCAGCAGCGAGAGTCACGAGTCCACTGAGA 607
SCPP1˙LW      ....ATTGAAGGTACTGAGTATAGTACGAGCAAC...AGCAGCAGCAGCAGCAGCAGCAGCAGCAGCGAAAGCACTGAGT 577
SCPP1˙PN      TGTGGTGGACAGCAACGAGACCAGTGAGAGCAGAAGCAGCAACAGCAGTAGCAGCAACGAGAGCCGAGAGTCCAGCCACA 601
SCPP1˙PP      ................................................................................ 171
SCPP1-C1˙CC   TATTGTTGACGGAACTGAGTACAGTACCAGCAACAGCAGCAGCAGCAGCAGCAGCAGCAGTAGCAGTGAAAGCACAGAGT 622
SCPP1-C2˙CC   TAGTGTTGATGGTACTGAGTACAGTACTAGCAACAGCAGCAGCAGCAGCAGCAGTAGCAGCAGCAGTGAAAGCACCGAGT 622
SCPP1-C1˙SR   TATTGTTGACGGTACTGAGTACAGTACTAGCAAT......AGCAGCAGCAGCAGCAGTAGCAGCAGTGAAAGCACCGAGT 478
SCPP1-C2˙SR   TAGTGTTGACGGTACTGAGTACAGTACCAGCAAT.........AGCAGCAGCAGCAGCAGTAGCAGTGAAAGCACTGAGT 613
SCPP1-C1˙SA   TATTGTTGACGGTACTGAGTACAGTACTAGCAAT.........AGCAGCAGCAGCAGCAGTAGCAGTGAAAGCACCGAGT 613
SCPP1-C2˙SA   TAGTGTTGACGGTACTGAGTACAGTACCAGCAAT......AGCAGCAGCAGCAGCAGTAGCAGCAGTGAAAGCACTGAGT 610
SCPP1-C1˙SG   TATTGTTGACGGTACTGAGTACAGTACTAGCAAT.........AGCAGCAGCAGCAGCAGTAGCAGTGAAAGCACCGAGT 595
SCPP1-C2˙SG   TAGTGTTGACGGTACTGAGTACAGTACCAGCAAT.........AGCAGCAGCAGCAGTAGCAGCAGTGAAAGCACCGAGT 559

               610  615  620  625  630  635  640  645  650  655  660  665  670  675  680  685
conservation  ***** **** **** ****** ***** ******* ******** *** ******************************
SCPP1˙CI      CCAAAAACAGCGCAGTGGATAGCGAAAGTCGCTCAACTGAGTGCCTTCCCGGGGCTGACAGCCAAGAATGTGACAGCGAG 687
SCPP1˙AM      CCAGCCACAGCACAGAGAGCACAGAGAGCCAGTCCAAAGAGTGCCCGCCCGGAACAGACAGCAACGAGTGCGACAGCGAC 417
SCPP1˙DR      CTAAAGACAGCGGCACGGATAGTGAAAGTCGCTCCACTGAGTGTGTGCCTGGCGATGACAGTCAAGATTGTGAGAGCGAG 693
SCPP1˙IP      CCACCGAGAGCACCGAAAGCAAACAGAGTCGCTCTAACGAGTGCCAGCCTGGAGCCGACAGCCAGGACTGCGACAGCGAC 687
SCPP1˙LW      CCAAAAGCAGTGCGATGGAGAGCGAAAGTCGCTCAACTGAGTGCCTTCCCGGGGCTGACAGCCAAGAATGTGACAGCGAG 657
SCPP1˙PN      GCACAGAGAGCAGGGAAAGCACAGAGAGCCGCTCGAAAGAGTGCCCGTCTGAGGCCGACAGCGATGAGTGCGACAGCGAT 681
SCPP1˙PP      ................................................................................ 171
SCPP1-C1˙CC   CCAAAAACAGTGCAGTGGACAGCGAAAGTCGCTTAAGTGAGTGCCAGCCAGGGGCTGACAGCCAAGAATGTGACAGCGAG 702
SCPP1-C2˙CC   CCAAAAACAACGCAGTGGACAGTGAAAGTCGCTCAGCTGAGTGCCAGCCAGGGGCTAACAGCCAAGAATGTGACAGCGAG 702
SCPP1-C1˙SR   CCAAAAACAGTGCAGTGGACAGCGAAAGTCGCTCAACTGAGTGCCAGCCAGGGGCTGACAGCCAAGAATGTGACAGCGAG 558
SCPP1-C2˙SR   CCAAAAACAGTGCAGTGGACAGCGAAAGCCGCTCAGCTGAGTGCCAGTCAGGGGCTGACAGCCAAGAATGTGACAGCGAG 693
SCPP1-C1˙SA   CCAAAGACAGCGCAGTGGACAGTGAAAGTCGCTCAATTGAGTGCCAGCCAGGGGCTGACAGCCAGGAATGTGACAGCGAG 693
SCPP1-C2˙SA   CCAAAAACAGTGCAGCAGACAGCGAAAGCCGCTCAGCTGAGTGCCAGCCAGAGGCTGACAGCCAAGAATGTGACAGCGAG 690
SCPP1-C1˙SG   CCAAACACAGCGCAGTGGACAGCGAAAGTCGCTCAACTGAGTGCCAGCCAGGGGCTGACAGCCAAGAATGTGACAGCGAG 675
SCPP1-C2˙SG   CCAAAAACAGTGCACCGGACAGCGAAAGCCGCTCAGCTGAGTGCCAGCCAGAGGCTGACAGCCAAGAATGTGACAGCGAG 639

               690        695  700  705  710  715  720  725  730  735  740  745  750  755  760
conservation  ******      ************************************************ *******************
SCPP1˙CI      GAAGAC......TTTCCTCAGGATATCGGTGATGATGGTGCAACCGACCCATTCAATGGCTTCCTAATGCCTGATGTCAC 761
SCPP1˙AM      GAATAC......CAGTTCCACGACGTCGGAGACGATGGAGCCACTGACCCCTTCAACGGCTTCCACACGACCGACAACGC 491
SCPP1˙DR      GAAAAC......CTTCCTCAGGATATCGGTGATGATGGAGCAACCGACCCTTTCAATGGCTTCTTAATGCCTGATGTCGC 767
SCPP1˙IP      AGCGACGAGTACGTCCTCCAGAACGTCGGAGACGACGGAACCAACGACCCATTTGACGGCTTCCACGTGCCTGACAGCAC 767
SCPP1˙LW      GAAGCC......TTTCCTCAGGATATCGGTGATGATGGTGCAACCGACCCTTTCAATGGCTTCCTAATGCCTGATGTCAC 731
SCPP1˙PN      GAATAC......GACTTCCACGACATCGGAGACGATGGAGCCAGTGAACCGCTGAACGGCTTCCGTACGCCCGAT..... 750
SCPP1˙PP      ................................................................................ 171
SCPP1-C1˙CC   GAAGAC......TTTCCTCAGGATATCGGTGATGATGGTGCAACCGACCCTTTCAATGGCATCCTAATGCCTGATGTCAC 776
SCPP1-C2˙CC   GAAGAC......TTTCCTCAGGATATCGGTGATGATGGTGAAACCGACCCTTTCAATGGCATCCTAATGCCTGATGTCAC 776
SCPP1-C1˙SR   GAAGAC......TTTCCTCAGGATATCGGTGATGATGGTGCAACCGACCCTTTCAATGGCATCCTAATGCCTGATGTCAC 632
SCPP1-C2˙SR   GAAGAC......TTTCCTCAGGATATCGGTGATGATGGTGCAACCGACCCTTTCAATGGCATCCTAATGCCTGATGTCAC 767
SCPP1-C1˙SA   GAAGAC......TTTCCTCAGGATATCGGTGATGATGGTGCAACCGACCCTTTCAATGGCATCCTAATGCCTGATGTCAC 767
SCPP1-C2˙SA   GAAGAC......TTTCCTCAGGATATCGGTGATGATGGTGCAACCGACCCTTTCAATGGCATCCTAATGCCTGATGTCAC 764
SCPP1-C1˙SG   GAAGAC......TTTCCTCAGGATATCGGTGATGATGGTGCAACGGACCCTTTCAATGGCATCCTAATGCCTGATGTCAC 749
SCPP1-C2˙SG   GAAGAC......TTTCCTCAGGATATCGGTGATGATGGTGCAACCGACCCTTTCAATGGCATCCTAATGCCTGATGTCAC 713
```

	765 . 770	
conservation	**********	
SCPP1˙CI	TGAGCCCTAG	771
SCPP1˙AM	C.........	492
SCPP1˙DR	TGAGCCCTAG	777
SCPP1˙IP	CGAG......	771
SCPP1˙LW	TGAGCCCTAG	741
SCPP1˙PN	....	750
SCPP1˙PP	....	171
SCPP1-C1˙CC	TGAGCCCTAG	786
SCPP1-C2˙CC	TGAGCCCTAG	786
SCPP1-C1˙SR	TGAGCCCTAG	642
SCPP1-C2˙SR	TGAGCCCTAG	777
SCPP1-C1˙SA	TGAGCCCTAG	777
SCPP1-C2˙SA	TGAGCCCTAG	774
SCPP1-C1˙SG	TGAGCCCTAG	759
SCPP1-C2˙SG	TGAGCCCTAG	723

X non conserved

X $\geq$ 55% conserved

X $\geq$ 85% conserved
