# Supplementary material for: A Genomic Survey of SCPP Family Genes in Fishes Provides Novel Insights into the Evolution of Fish Scales
Source: Int J Mol Sci. 2017 Nov 16;18(11):2432. doi: 10.3390/ijms18112432 (PMC5713400; doi:10.3390/ijms18112432)
Supplement: Supplementary file 1 [file ijms-18-02432-s001.zip › ijms-236743-supplementary/Figure S8.ODAM.cds.pdf]

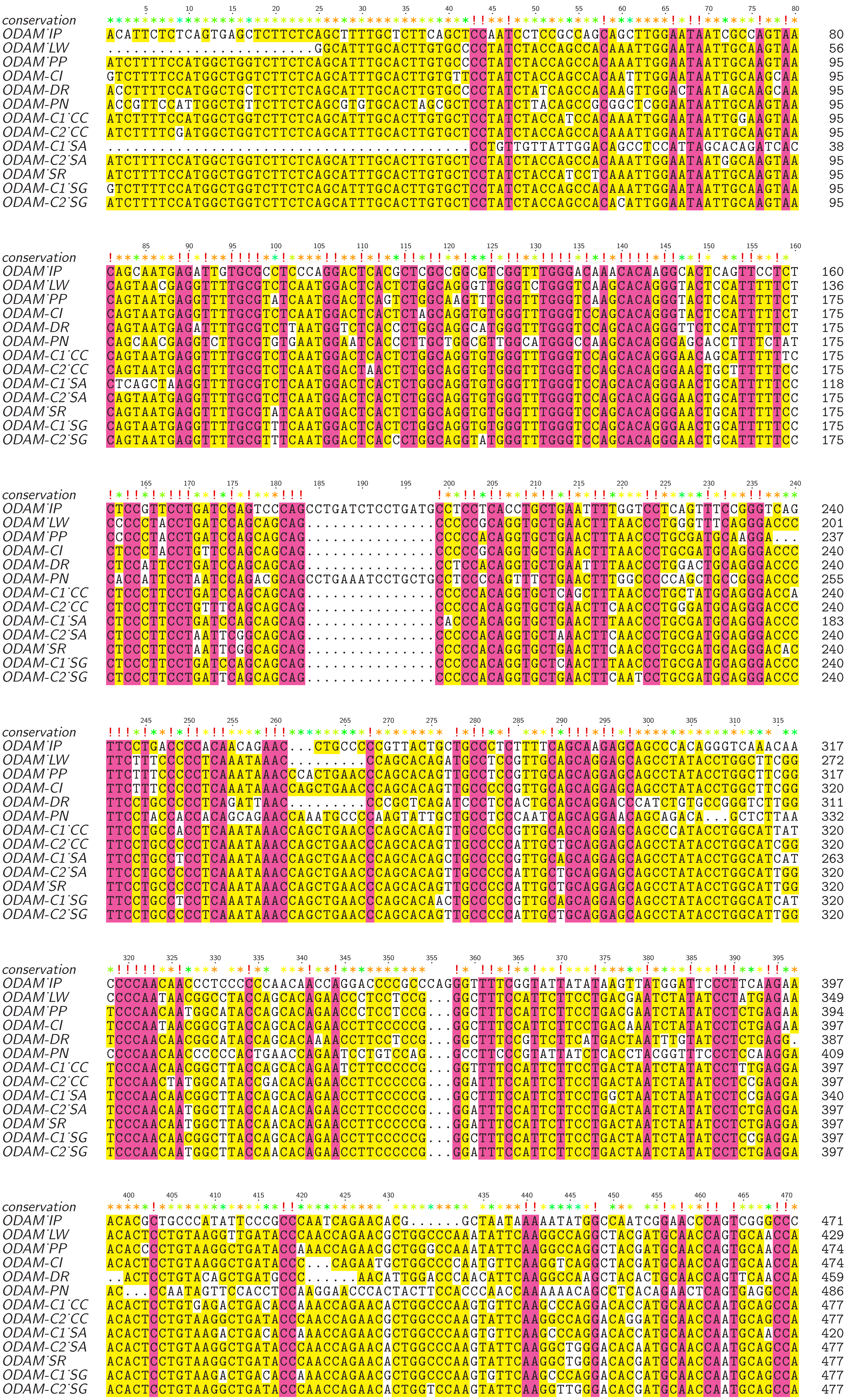
conservation
ODAM_IP
ODAM_LW
ODAM_PP
ODAM-CI
ODAM-DR
ODAM-PN
ODAM-C1_CC
ODAM-C2_CC
ODAM-C1_SA
ODAM-C2_SA
ODAM_SR
ODAM-C1_SG
ODAM-C2_SG

```
                 475   480   485   490   495                               500  505  510  515
conservation   *****   *** **!!********* *** * ** *  *                       *    *        ***
ODAM_IP       ACTAAA...GTCCCTCAGCCTGTGCAGAAT.................................AACGGGAAGGTGGTGGA   515
ODAM_LW       ATGCAA...CCAGTGCAACCAATACAGGGTTAGGGTAGGATTAGGAAC...............AGGGTGAAGGCAGTAGA   491
ODAM_PP       ATGCAA...CCAGTGCAACCAATTCGACCAATACAGAAC........................AGGGTGAAGGCAGTAGA   527
ODAM-CI       GTGCAA...CCAATGCAACCAGTGCAACCAGTGCAACCAATCCAGAAC...............AGGGTGAAGGCAGTAGA   536
ODAM-DR       ATGCAA...CCAATGCAGCCAATGCAACCAATACAGCCAATACAACCAATCCAGAAC......AGAGGGAATTTGGCAGA   530
ODAM-PN       ACTCCAGCACCCCCACAGCCTGCGCAGCCAGTAAATATTAAACGGTTCTCAAGAAACTCAATAAAGGGAAACCTTCCTCC   566
ODAM-C1_CC    GTGCAA...CCAATCCAGAAAATATTTCACATTTTTCAG........................AACAGGGGGAAAGCAGA   530
ODAM-C2_CC    GTGCAA...CCATTGCAACCAATGCAGCCAGTGCAACCAATGCAACCA...............AGCCAGGGAAAGTCAGA   539
ODAM-C1_SA    ATGCAG...CCAGTGCAACCAATCCAGCCTCTGCCACCAAACAACAAT................................   465
ODAM-C2_SA    GTGCAA...CCAATGCAACCAATGCAACCAATGCAGCCA........................AACAGGGGAAAGTCAGA   530
ODAM_SR       GTGCAA...CCAATGCAACCAATGCAGCCAGTGCAACCAATGCAACCAATGCAACCA..................ATACA   536
ODAM-C1_SG    GTGCAA...CCAATACAACCAATCCAG.....................................................   501
ODAM-C2_SG    GTGCAA...CCAATGCAGCCAGTGCAACCAATGCAACCAATGCAACCAATACAACCAATCCAGAACAGGGGAAAGTCAGA   554

                 520   525   530   535   540   545   550   555   560   565   570
conservation  ****   ******************************************** ***
ODAM_IP       GCCGTGGCACATGACTCCGCCCCCTGAAAACCGTGGTGATCGTCCTGGGCGTGGT   570
ODAM_LW       CCCG...ATGGTAACGTCTGCTCCTGATTACCGTGGTGATCGGCCCGGTCCTGGA   543
ODAM_PP       CCCG...ATGGTAACGTCTGCTCCTGATTACCGTGGTGATCGGCCCGGTCCTGGA   579
ODAM-CI       CCCG...AAGGTAACGTCTGCTCCTGATTACCGTGGTGATCGGCCCGGTCCTGGA   588
ODAM-DR       CCCG...AAAGCAACGTCTGTGCCTGAATACCGGGGTGATCGACCCGGTCCTGGA   582
ODAM-PN       TCCT...........................GGTGGCCAGCGTCCTCCACTTGGC   594
ODAM-C1_CC    CCCG...AAGGTAACGTCTGCTCCTGATTACCGTGGTGATCGGCCCGTTCCTGGA   582
ODAM-C2_CC    CCAG...AAGGTAACGTCTGCTCCTGATTACCGTGGTGATCGGCCTGGTCCGGGA   591
ODAM-C1_SA    .......................................................   465
ODAM-C2_SA    CCCG...AAGGTAACGTCTGCTCCTGATTACCGTGGTGATCGGCCCGGTCCGGGA   582
ODAM_SR       ACCAATCCAGGTAACGTCTGCTCCTGATTACCGTGGTGATCGGCCCGGTCCGGGA   591
ODAM-C1_SG    .......................................................   501
ODAM-C2_SG    CCCG...AAGGTAACGTCTGCTCCTGATTACCGTGGTGATCGGCCCGGTCCGGGA   606
```

X non conserved

X ≥ 55% conserved

X ≥ 85% conserved
