# Supplementary material for: A Genomic Survey of SCPP Family Genes in Fishes Provides Novel Insights into the Evolution of Fish Scales
Source: Int J Mol Sci. 2017 Nov 16;18(11):2432. doi: 10.3390/ijms18112432 (PMC5713400; doi:10.3390/ijms18112432)
Supplement: Supplementary file 1 [file ijms-18-02432-s001.zip › ijms-236743-supplementary/Table S1&S2.pdf]

International Journal of
Molecular Sciences

MDPI

Article

A Genomic Survey of *SCPP* Family Genes in Fishes Provides Novel Insights into the Evolution of Fish Scales

Yunyun Lv, Kazuhiko Kawasaki, Jia Li, Yanping Li, Chao Bian, Yu Huang, Xinxin You and Qiong Shi

Table S1. Accession numbers of zebrafish *SPARCL1* and *SCPP* genes.

zebrafish	mRNA	Protein
SPARCL1	NM_001130605.1	NP_001124077.1
SPP1	AY620817.1	AAT39545.1
SCPP1	EU642610.1	ACF33438.1
ODAM	EU642608.1	ACF33436.1
fa93e10	NM_001145556.1	NP_001139028
SCPP5	EU642611.1	ACF33439.1
SCPP7	EU642613.1	ACF33441.1
SCPP6	EU642612.1	ACF33440.1
SCPP9	EU642615.1	ACF33443.1

Table S2. Accession numbers of the ten fish genomes used for this study.

Organism	Accession
Astyanax mexicanus	GCF_000372685.1
Ctenopharyngodon idellus	http://www.ncgr.ac.cn/grasscarp/
Cyprinus carpio	GCA_001270105.1
Ictalurus punctatus	GCF_001660625.1
Leuciscus waleckii	GCA_900092035.1
Pimephales promelas	GCA_000700825.1
Pygocentrus nattereri	GCF_001682695.1
Sinocyclocheilus anshuiensis	GCF_001515605.1
Sinocyclocheilus grahamin	GCF_001515645.1
Sinocyclocheilus rhinocerous	GCF_001515625.1
