# Supplementary material for: A Genomic Survey of SCPP Family Genes in Fishes Provides Novel Insights into the Evolution of Fish Scales
Source: Int J Mol Sci. 2017 Nov 16;18(11):2432. doi: 10.3390/ijms18112432 (PMC5713400; doi:10.3390/ijms18112432)
Supplement: Supplementary file 1 [file ijms-18-02432-s001.zip › ijms-236743-supplementary/Figure S1.SPARCl1.cds.pdf]

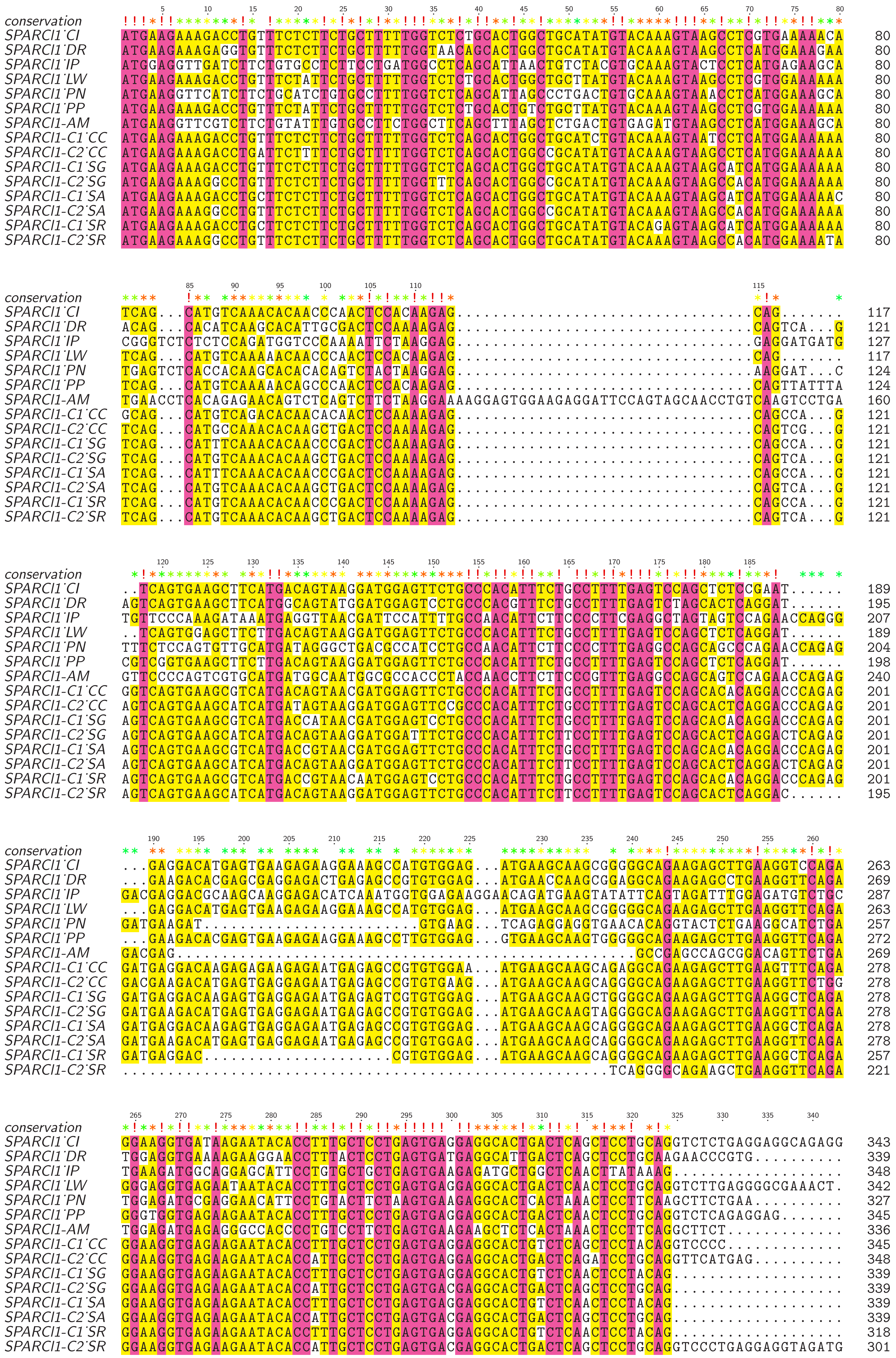
conservation
SPARCl1_CI
SPARCl1_DR
SPARCl1_IP
SPARCl1_LW
SPARCl1_PN
SPARCl1_PP
SPARCl1-AM
SPARCl1-C1_CC
SPARCl1-C2_CC
SPARCl1-C1_SG
SPARCl1-C2_SG
SPARCl1-C1_SA
SPARCl1-C2_SA
SPARCl1-C1_SR
SPARCl1-C2_SR

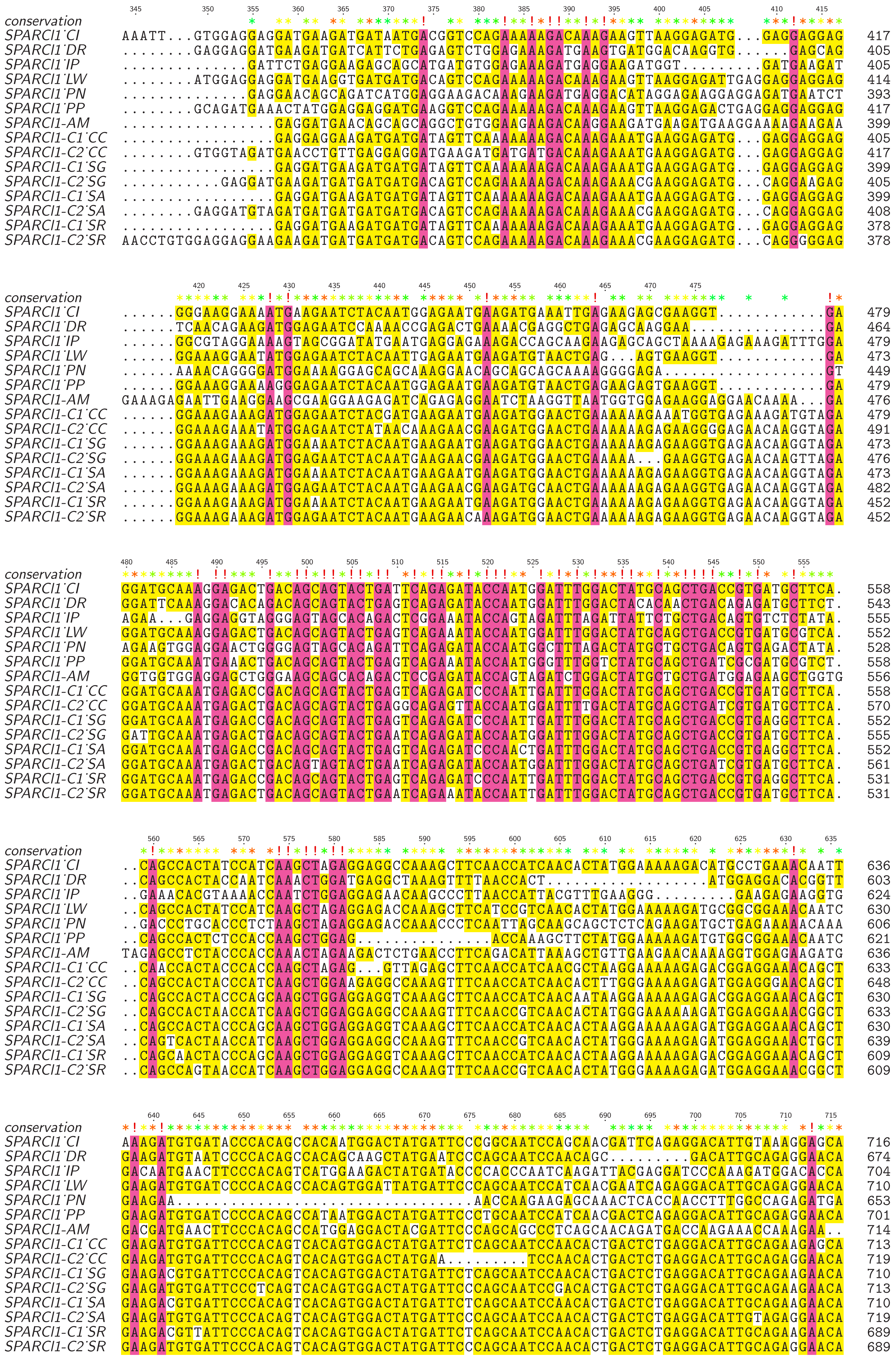
conservation
SPARCl1¨CI
SPARCl1¨DR
SPARCl1¨IP
SPARCl1¨LW
SPARCl1¨PN
SPARCl1¨PP
SPARCl1-AM
SPARCl1-C1¨CC
SPARCl1-C2¨CC
SPARCl1-C1¨SG
SPARCl1-C2¨SG
SPARCl1-C1¨SA
SPARCl1-C2¨SA
SPARCl1-C1¨SR
SPARCl1-C2¨SR

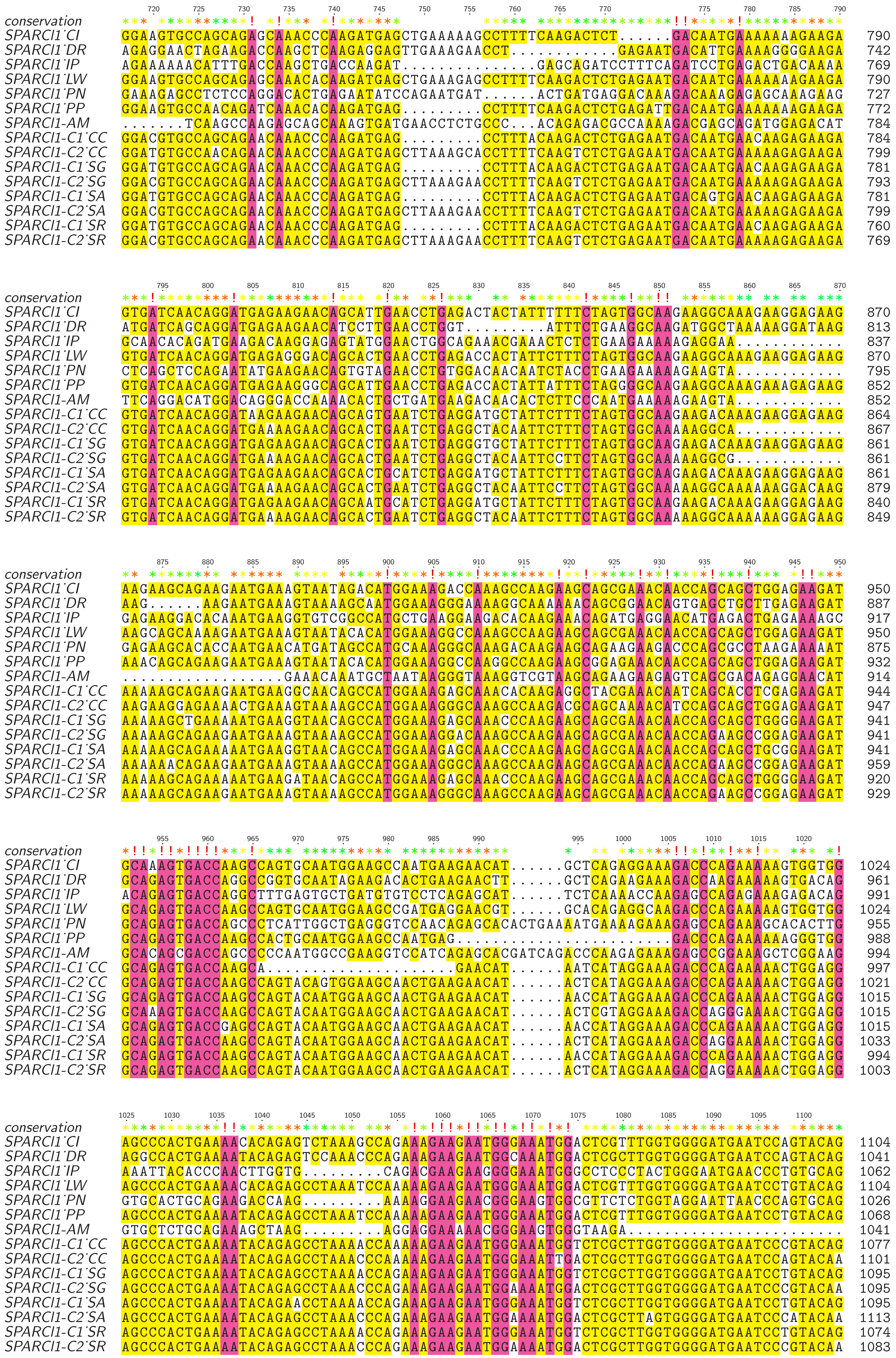

```
             1105      1110      1115      1120      1125      1130              1135      1140      1145      1150      1155      1160      1165      1170      1175
conservation
SPARCl1˙CI     ATTAGGGCCACTATGGAGCTTTACCCTGAC......ATTCGACCTACCCATCGGCCCAGTGGACAGGGGGTGCCAGCTGA 1178
SPARCl1˙DR     ATTAGGGCCACTATGGATCTTTACCCAGAT......GTTCGCCTCACCCGTCACTCCAATGGCAGGGGGTGCCAGCTGA 1115
SPARCl1˙IP     TTCAGGGCTGCAGTGGATCTTTTCCCCAAA......AAGAATTTCACTGTGCATATGTACATGTAATCATTATATCCAGA 1136
SPARCl1˙LW     ATTAGGGCCACTATGGAGCTTTACCCTGAC......ATTCGGCCCACACATCGGCCCAGCAGACAGGGGGAGCCAGCTGA 1178
SPARCl1˙PN     ATTCGGGCAACAGTGGAGCTGTTCCCCAGACCTGTGGTCAGGTCCAGACTCGGAGCCGACGGCCAGGAAGCTCCCGCCGA 1106
SPARCl1˙PP     ATTAGGGCCACTATGGAGCTTTACCCTGAC......ATTCGGCCCAGCAGA............CAGGGGGAGCCAGCTGA 1130
SPARCl1-AM     ................................................................................ 1041
SPARCl1-C1˙CC  ATCAGGGCCGCTATGGAGCTTTACCCTGAC......ATTCGACCCACCCATCGGCCCAGCGGACAGGGGTTGTCAGATGA 1151
SPARCl1-C2˙CC  ATTAGGGCCACTATGGAGCTTTACCCTGAC......ATTCGACCCACCCGTTGGCCCAGTGGACAGGGGTTACCAGTTGA 1175
SPARCl1-C1˙SG  ATCAGGGCCGCTATGGAGCTTTACCCTGAC......ATTCGACCCACCCATCGGCCCAGTGGACAGGGGTTGCCAGATGA 1169
SPARCl1-C2˙SG  ATTAGGGCCACTATGGAGCTTTACCCTGAC......ATTCGACCCACCCATCGGCCCAGTGGACAGGGGTTGCCAGCTGA 1169
SPARCl1-C1˙SA  ATCAGGGCCGCTATGGAGCTTTACCCTGAC......ATTCGACCCACCCATCGGCCCAGTGGACAGGGGTCGTCAGATGA 1169
SPARCl1-C2˙SA  ATTAGGGCCACTATGGAGCTTTACCCTGAC......ATTCGACCCACCCATCGGCCCAGTGGACAAGGGTTGCCAGCTGA 1187
SPARCl1-C1˙SR  ATCAGGGCCGCTATGGAGCTTTACCCTGAC......ATTCGACCCACCCATCGGCCCAGTGGACAGGGGTTGCCAGATGA 1148
SPARCl1-C2˙SR  ATTAGGGCCACTATGAAGCTTTACCCTGAC......ATTCGACCCACCCATCGGCACAGTGGACAGGGGTTGCCAGCTGA 1157

               1180      1185      1190      1195      1200      1205      1210      1215      1220      1225      1230      1235      1240      1245      1250      1255
conservation
SPARCl1˙CI     CCCCTGTGAGAACTTTCGCTGTAAAAGAGGGAAGACATGCAAAATAAATGATGAGAACAAACCCGTCTGTGTGTGTCAGG 1258
SPARCl1˙DR     CCCCTGTGAAAATTTCCGCTGCAAAAGAGGGAAAACTTGCAAACTGAACGATGAGAACAAGCCCCTCTGTGTGTGTCAGG 1195
SPARCl1˙IP     CTCATGTGAGAACTTCCACTGTAAGCGAGGGAAGACATGCAAGCTGAACGATATGAAGAAGCCCAGCTGTGTGTGTCAGG 1216
SPARCl1˙LW     CCCTTGTGAGAACTTTCGCTGTAAAAGAGGAAAGACATGCAAAATGAACGATGAGAACAAGCCCGTCTGTGTGTGTCAGG 1258
SPARCl1˙PN     CCCATGTGAGAACTTCCACTGTAAGCGAGGGAAGACATGCAAACTGAACGAAGAACAGAAGCCCAACTTGTGTGTGTCAAG 1186
SPARCl1˙PP     CCCCTGTGAGAACTTTCGCTGTAAAAGAGGAAAGACATGCAAAATGAACGATGAGAACAAGCCCGTCTGTGTGTGTCAGG 1210
SPARCl1-AM     ................................................................................ 1041
SPARCl1-C1˙CC  CCCCTGCGAGAACTTTCGCTGTAAAAGAGGAAAGACGTGCAAAATGAATGATGAGAACAAACCTGTCTGTGTGTGTCAGG 1231
SPARCl1-C2˙CC  CCCCTGTGAGAACTTTCGCTGTAAAAGAGGGAAGACATGCAAAATGAACGATGAGAACAAGCCCGTCTGTGTGTGTCAGG 1255
SPARCl1-C1˙SG  CCCCTGTGAGAACTTTCGCTGTAAAAGAGGAAAAACATGCAAAATGAATGATGAGAACAAACCCGTCTGTGTGTGTCAGG 1249
SPARCl1-C2˙SG  CCCCTGCGAGAACTTTCGCTGTAAAAGAGGGAAGACATGCAAAATGAACGATGACAACAAGCCCGTCTGTGTGTGTCAGG 1249
SPARCl1-C1˙SA  CCCCTGCGAGAACTTTCGCTGTAAAAGAGGAAAGACATGCAAAATGAATGATGAGAACAAACCCGTCTGTGTGTGTCAGG 1249
SPARCl1-C2˙SA  CCCCTGCGAGAGCTTTCGCTGTAAAAGAGGGAAGACATGCAAAATGAACGATGAGTACAAGCCCGTCTGTGTGTGTCAGG 1267
SPARCl1-C1˙SR  CCCCTGCGAGAACTTTCGCTGTAAAAGAGGAAAGACATGCAAAATGAATGATGAGAACAAACCCGTCTGTGTGTGTCAGG 1228
SPARCl1-C2˙SR  CCCCTGCGAGAACTTTCGCTGTAAAAGAGGGAAGACATGCAAAATGAACGAGGAGAACAAGCCCGTCTGTGTGTGTCAGG 1237

               1260      1265      1270      1275      1280      1285      1290      1295      1300      1305      1310      1315      1320      1325      1330      1335
conservation
SPARCl1˙CI     AGCCGTCAGAATGTCCCCCCAGTGTAAACGATTTTGACCATGTATGCGGAACTGACAATAAGACATATGACACATCCTGT 1338
SPARCl1˙DR     AGCCGACAGAATGTCCTCCTAATGTAAACGATTTTGAACATGTCTGCGGAACTGACAATAAGACATATGACTCATCTTGT 1275
SPARCl1˙IP     ATCCTGCAGACTGTCCTCCAGGTCTCAGTGAGTTTGACCATGTGTGTGGAACAGATAACCAGACTTATGACTCATACTGC 1296
SPARCl1˙LW     AGCCGTCAGAATGTCCTCCCAGTGTAAATGATTTTGACCATGTTTGCGGAACTGACAATAAGACATATGACACATCCTGT 1338
SPARCl1˙PN     AACCTGCAGACTGTCCTCCAAGTGTCGCTGAATTTGAACATGTGTGTGGAACAGACAACCAGACATACGACACCTCCTGC 1266
SPARCl1˙PP     AGCCGTCAGAATGTCCTCCCAGTGTAAATGATTTTGACCATGTTTGCGGAACTGACAATAAGACATATGACACATCCTGT 1290
SPARCl1-AM     ................................................................................ 1041
SPARCl1-C1˙CC  AGCCGTCAGAATGTCCTCTCAGTGTAAATGATTTTGAGCATGTATGTGGAACTGACAATAAGACATATGACACATCCTGT 1311
SPARCl1-C2˙CC  AGCCGTCAGAATGTCCTCCCAGTGTAAATGATTTAGACCATGTATGCGGAACAGACAATAAGACATATGACACATCCTGT 1335
SPARCl1-C1˙SG  AGCCATCAGAATGTCCTACCAGTGTAAATGATTTTGAGCATGTATGTGGAACTGACAATAAGACATATGACACATCCTGT 1329
SPARCl1-C2˙SG  AGCCGTCAGAATGTCCTCCCAGTGTAAATGATTTAGACCATGTATGCGGAACTGACAATAAGACATATGACACATCCTGT 1329
SPARCl1-C1˙SA  AGCCGTCAGAATGTCCTACCAGTGTAAATGATTTTGAGCATGTATGCGGAACTGACAATAAGACATATGACACATCCTGT 1329
SPARCl1-C2˙SA  AGCCGTCAGAATGTCCTCCCAGTGTAAATGATTTAGACCATGTATGCGGAACTGACAATAAGACATATGACACATTCTGT 1347
SPARCl1-C1˙SR  AGCCGTCAGAATGTCCTACCAGTGTAAATGATTTTGAGCATGTATGCGGAACTGACAATAAGACATATGACACATCCTGT 1308
SPARCl1-C2˙SR  AGCCGTCAGAATGTCCTCCCAGTGTAAATGATTTAGACCATGTATGCGGAACTGACAATAAGACATATGACACATCCTGT 1317

               1340      1345      1350      1355      1360      1365      1370      1375      1380      1385      1390      1395      1400      1405      1410      1415
conservation
SPARCl1˙CI     CAGCTCTTTGCCACGAAGTGTGGTCTTGATGGAACCAAAATAGGCCACAGACTTCACCTGGACTACACTGGATCCTGTAA 1418
SPARCl1˙DR     CACCTTTTTGCTACCAAGTGTGGTCTTGAGGGAACCAAATTAGGTCACAGGCTTCATCTGGACTACACTGGATCATGCAA 1355
SPARCl1˙IP     CAGCTTTTTGCCATCAAGTGCAGCCTTGAGGGCAGCAAGAAAGGGCACAGACTACATCTGGACTACAGCGGCTCCTGCAA 1376
SPARCl1˙LW     CAGCTCTTTGCCACCAAGTGTAGCCTTGACGGGACCAAAATAGGCCACAGACTTCACCTGGACTACACTGGGTCCTGCAA 1418
SPARCl1˙PN     CAGCTCTTTGCCACCAAGTGTGGCCTTGAGGGCACCAAGAAAGGTCACAAACTGCACCTGGACTACACAGGCTCCTGCAA 1346
SPARCl1˙PP     CAGCTCTTTGCCACCAAGTGTAGTCTTGACGGGACCAAAGTAGGCCACAGACTTCACCTGGACTACTCTGGGTCCTGCAA 1370
SPARCl1-AM     ................................................................................ 1041
SPARCl1-C1˙CC  CAGCTTTTTGCCACCAAGTGTGGCCTTGAGGGGACCAAAATAGGCCACAGACTTCACCTGGATTACACTGGATCCTGCAA 1391
SPARCl1-C2˙CC  CAGCTCTTTGCAACCAAGTGTGGCCTTGAGGGGACAAAAATAGGCCACAGACTTCACCTGGAATACACTGGATCCTGCAA 1415
SPARCl1-C1˙SG  CAGCTTTTTGCCACCAAGTGTGGCCTTGAGGGGACCAAAATAGGCCACAGACTTCACCTGGATTACACTGGATCCTGCAA 1409
SPARCl1-C2˙SG  CAGCTCTTTGCCACCAAGTGTGGCCTTGAGGGGACCAAAATAGGCCACAGACTTCACCTGGATTACACTGGATCCTGCAA 1409
SPARCl1-C1˙SA  CAGCTTTTTGCCACCAAGTGTGGCCTTGAGGGGACCAAGATAGGCCACAGACTTCACCTGGATTACACTGGATCCTGCAA 1409
SPARCl1-C2˙SA  CAGCTCTTTGCCACCAAGTGTGGCCTTGAGGGGACCAAAATAGGCCACAGACTTCACCTGGATTACACTGGATCCTGCAA 1427
SPARCl1-C1˙SR  CAGCTTTTTGCCACCAAGTGTGGCCTTGAGGGGACCAAAATAGGCCACAGACTTCACCTGGATTACACTGGATCCTGCAA 1388
SPARCl1-C2˙SR  CAGCTCTTTGCCACCAAGTGTGGCCTTGAGGGGACCAAAATAGGCCACAGACTTCACCTGGATTACACTGGATCCTGCAA 1397

               1420      1425      1430      1435      1440      1445      1450      1455      1460      1465      1470      1475      1480      1485      1490      1495
conservation
SPARCl1˙CI     ATTTATTGCTCCATGTCTAGAGTCAGAACTGGTGCAGTTCCCTCTGCGCATGCGGGACTGGCTGAAGAACGTGCTTCTGC 1498
SPARCl1˙DR     ATTTATTGCTCCATGTGTGGAGTCAGAGCTGGTGCAGTTCCCACTGCGAATGCGGGACTGGCTGAAGAACGTGCTTCTGC 1435
SPARCl1˙IP     ATTTATTCCACCATGTCTGAAGACAGAGCTGATACATTTTCCACTGCGCATGAGAGACTGGCTGAAGAATGTGCTGCTGC 1456
SPARCl1˙LW     ATTTATTGCTCCATGTCTAGAGTCAGAGCTGGTGCAGTTCCCTCTACGTATGCGGGATTGGCTGAAGAATGTGCTTCTGC 1498
SPARCl1˙PN     ATTTATCCCACCATGCCTGAGCACAGAACTTGTGCAGTTTCCACTTCGTATGAGGGACTGGCTGAAGAACGTGCTGCTGC 1426
SPARCl1˙PP     ATATATA......TACCTATTGGTGTTTTAGATGTATTTTCCAAAGAGATCTCAACAATGTCTTAGAAATTACATTTTGG 1444
SPARCl1-AM     ................................................................................ 1041
SPARCl1-C1˙CC  ATTTATTCCTCCATGTCCAGAGTCAGAGCTGGTGCAGTTCCCTCTGCGTATGCGAGATTGGCTGAAGAATGTGCTTTTGC 1471
SPARCl1-C2˙CC  ATTTATTCCTCCATGTCCAGAGTCGGAGCTGGTGCAGTTCCCTCTGCGTATGCGGGATTGGCTGAAGAACGTGCTTCTGC 1495
SPARCl1-C1˙SG  ATTTATTCCTCCATGTCCAGAGTCGGAGCTGGTGCAGTTCCCTCTGCGTATGCGAGATTGGCTGAAGAATGTGCTTTTGC 1489
SPARCl1-C2˙SG  ATTTATTCCTCCATGTCCAGAGTCGGAGCTGGTGCAGTTCCCTCTGCGTATGCGGGATTGGCTGAAGAATGTGCTTCTGC 1489
SPARCl1-C1˙SA  ATTTATCCTTCCATGTCCAGAGTCGGAGCTGGTGCAGTTCCCTCTGCGTATGCGAGATTGGCTGAAGAACGTGCTTTTGC 1489
SPARCl1-C2˙SA  ATTTATTCCTCCATGTCCAGAGTCGGAGCTGGTGCAGTTCCCTCTGCGTATGCGGGATTGGCTGAAGAACGTGCTTCTGC 1507
SPARCl1-C1˙SR  ATTTATTCCTCCATGTCCAGAGTCGGAGCTGGTGCAGTTCCCTCTGCGTATGCGAGATTGGCTGAAGAACGTGCTTTTGC 1468
SPARCl1-C2˙SR  ATTTATTCCTCAATGTCCAGAGTCGGAGCTGGTGCAGTTCCCTCTGCGTATGCGGGATTGGCTGAAGAACGTGCTTCTGC 1477
```

```
             1500      1505      1510      1515      1520      1525      1530      1535      1540      1545      1550      1555      1560      1565      1570      1575
conservation
SPARCl1˙CI    AGTTATATGAGCATGACTCCATGTCTCCTGGCTTTCTCACAGCTAAACAAAGAATCAGGGTTCAGAAGATTTATGAGAGT  1578
SPARCl1˙DR    AGTTATATGAGCATGACTCCATGTCTCCTGGCTTTCTCACAGCTAAACAGAGAATCAGGGTTCAGAAGATCTATGAGAGC  1515
SPARCl1˙IP    AGCTCTACGAACAAGAC...............TTCCTTACAGCCAAGCAGAGATCCAGAGTTCAGAAGATGTACGAGAAT  1521
SPARCl1˙LW    AGTTATACGAGCATGACTCCATGTCTCCTGGCTTTCTCACAGCTAAACAAAGAATCAGGGTTCAGAAGATCTATGAGAGC  1578
SPARCl1˙PN    AGCTCTATGAGCACGACTCAGTGACTCCTGGCTTCCTCACGGCCAAGCAGAGAGCCAGGGTTCAGAAGCTGTATGAGAAT  1506
SPARCl1˙PP    TTTAGCAAGACACAAAGTCTCAGAGGAGGCAG...ATGAAACTATGGAGGAGGATGAAGGTCCAGAAAAAGACAAAGAAG  1521
SPARCl1-AM    ................................................................................  1041
SPARCl1-C1˙CC AGTTATATGAGCATGAATCCATGTCTCCTGGCTTTCTCACAGCTAAACAAAGTATCAGGGTTCAGAAGATCTATGAGAGT  1551
SPARCl1-C2˙CC AGTTATATGAGCATGAATCCATGTCTCCTGGCTTTCTCACAGCTAAACAAAGAATCAGGGTTCAGAAGATCTATGAGAGT  1575
SPARCl1-C1˙SG AGTTATATGAACATGAATCCATGTCTCCTGGCTTTCTCACAGCTAAACAAAGTATCAGGGTTGAGAAGATCTTTGAGAGT  1569
SPARCl1-C2˙SG AGTTATATGAGCATGAATCCATGTCTCCTGGCTTTCTCACAGCTAAACAAAGAATCAGGGTTCAGAAGATCTATGAGAGT  1569
SPARCl1-C1˙SA AGTTATATGAACATGAATCCATGTCTCCTGGCTTTCTCACAGCTAAACAAAGTATCAGGGTTCAGAAGATCTTTGAGAGT  1569
SPARCl1-C2˙SA AGTTATATGAGCATGAATCCATGTCTCCTGGCTTTCTCACAGCTAAACAAAGAATCAGGGTTCAGAAGATCTATGATAGT  1587
SPARCl1-C1˙SR AGTTATATGAACATGAATCCATGTCTCCTGGCTTTCTCACAGCTAAACAAAGTATCAGGGTTCAGAAGATCTTTGAAAGT  1548
SPARCl1-C2˙SR AGTTATATGAGCATGAATCCATGTCTCCTGGCTTTCTCACAGCTAAACAAAGAATCAGGGTTCAGAAGATCTATGAGAGT  1557

             1580      1585      1590      1595      1600      1605      1610      1615      1620      1625      1630      1635      1640      1645      1650      1655
conservation
SPARCl1˙CI    GAGAGGCGTCTTCATGCAGGCGATCACACCGCCGAGCTTCTACAGCAGGACTTTGAGAAGAACTACAACATGTACATCTA  1658
SPARCl1˙DR    GAGAGACGTCTTCATGCAGGAGATCACCCTGTTGAAATTCTGCAGCAGGACTTTGAGAAGAACTACAACATGTACATCTA  1595
SPARCl1˙IP    GAGAGGCGTCTCCATGCAGGCGATCACCCAGTTGAGCTCTTGGTTCGGGACTTTGAAAAAGAATTACAACATGTACATATA  1601
SPARCl1˙LW    GAGAGGCGTCTTCATGCAGGCGATCACCCCGTCTAGCTTCTACAGCAGGACTTTGAGAAGAACTACAACATGTACATCTA  1658
SPARCl1˙PN    GAGCGGCGTCTCCATGCAGGGGACCATTCCATTGAATTGCTGGCTCAGGACTTTGAGAAAAACTACCCTATGTACATCTA  1586
SPARCl1˙PP    TTAGGAGACTGAGG.........AGGAGGAGGGAAAGGAAAAAGGGAGAATCTACAATGGAGAATGAAGATGTAACTGAG  1592
SPARCl1-AM    ................................................................................  1041
SPARCl1-C1˙CC GAGAGACGTCTTCATGCAGACGATCACCCCGTTGAGATTCTACAGCAGGACTTTGAGAAGAACTACAACATGTACATCTA  1631
SPARCl1-C2˙CC GAGAGACGCCTTCATGCAGGCGATCACCCTGTTGAACTTCTACAGCAGGACTTTGAGAAGAACTACAACATGTACATCTA  1655
SPARCl1-C1˙SG GAGAGACGTCTTCATGCAGGCGATCACCCCGTTGAGATTCTACAGCAGGACTTTGAGAAGAACTACAACATGTACATCTA  1649
SPARCl1-C2˙SG GAGAGACGGCTTCATGCAGGCGATCACCCTGTTGAACTTCTACAGCAGGACTTTGAGAAGAACTACAACATGTACATCTA  1649
SPARCl1-C1˙SA GAGAGATGTCTTCATGCAGGCGATCACCCTGTTGAGATTCTACAGCAGGACTTTGAGAAGAACTACAACATGTACATCTA  1649
SPARCl1-C2˙SA GAGAGACGGCTTCATGCAGGCGATCACCCTGTTGAACTTCTACAGCAGGACTTTGAGAAGAACTACAACATGTACATCTA  1667
SPARCl1-C1˙SR GAGAGACGTCTTCATGCAGGTGATCACCCCGTTGAGATTCTACAGCAGGACTTTGAGAAGAACTACAACATGTACATCTA  1628
SPARCl1-C2˙SR GAGAGACGGCTTCATGCAGGCGATCACCCTGTTGAACTTCTACAGCAGGACTTTGAGAAGAACTACAACATGTACATCTA  1637

             1660      1665                1670      1675      1680      1685      1690      1695      1700      1705      1710      1715      1720      1725
conservation
SPARCl1˙CI    TCCAGTG.........CACTGGCAGTTTGCACAAATGGACCAACATCCTTCAGACAGGTTTCTTACTCACTCTGAGCTGG  1729
SPARCl1˙DR    TCCAGTG.........CACTGGCAGTTTGCACAGATGGACCAGCATCCTTCAGACAGGTTTTTGACTCACTCTGAGCTGG  1666
SPARCl1˙IP    CCCAGTG.........CATTGGCAGTTTGCACAAATGGACCAACATCCATCAGACAGGTTCCTGTCTCACTCAGAGCTTG  1672
SPARCl1˙LW    TCCAGTG.........CACTGGCAGTTTGCACAAATGGACCAACATCCTTCAGACAGGATTCTTTCTCACTCTGAGCTGG  1729
SPARCl1˙PN    TCCTGTC.........CACTGGCAGTTCGCGCAGATGGATCAGCATCCATCAGACAGGTTTCTGTCTCACTCGGAGCTGG  1657
SPARCl1˙PP    AAGAGTGAAGATATCTCACAGGAAAATAGCTTCTATGGAAAATTCTTAAAAGTGTAAGATTCTTTCTCACTCTGAGCTGG  1672
SPARCl1-AM    ................................................................................  1041
SPARCl1-C1˙CC TCCAGTG.........CACTGGCAGTTTGCACAGATGGACCAGCATCCTTCAGACAAGTTTCTTTCTCACTCTGAGCTGG  1702
SPARCl1-C2˙CC TCCAGTT.........CACTGGCAGTTTGCACAGATGGACCAGCATCCTTCAGACAGGTTTCTTTCTCATTCGGAGCTGG  1726
SPARCl1-C1˙SG TCCAGTG.........CACTGGCAGTTTGCACAGATGGACCAGCATCCTTCAGACAAGTTTCTTTCTCACTCTGAGCTGG  1720
SPARCl1-C2˙SG TCCAGTT.........CACTGGCAGTTTGCACAGATGGACCAACATCCTTCAGACAGGTTTCTTTCTCATTCTGAGCTGG  1720
SPARCl1-C1˙SA TCCAGTG.........CACTGGCAGTTTGCACAGATGGACCAGCATCCTTCAGACAAGTTTCTTTCTCACTCTGAGCTGG  1720
SPARCl1-C2˙SA TCCAGTT.........CACTGGCAGTTTGCACAGATGGACCAACATCCTTCAGACAGGTTTCTTTCTCATTCTGAGCTGG  1738
SPARCl1-C1˙SR TCCAGTG.........CACTGGCAGTTTGCACAGATGGACCAGCATCCTTCAGACAAGTTTCTTTCTCACTCTGAGCTGG  1699
SPARCl1-C2˙SR TCCAGTT.........CACTGGCAGTTTGCACAGATGGACCAACATCCTTCAGACAGGTTTCTTTCTCATTCTGAGCTGG  1708

             1730      1735      1740      1745      1750      1755      1760      1765      1770      1775      1780      1785      1790      1795      1800      1805
conservation
SPARCl1˙CI    CTCCGCTGCGCGTTCCACTGGTTCCCATGGAGCATTGCACCTCAGTCTTTTTCCAAAAGTGTGATGCCGACAAGGACAAA  1809
SPARCl1˙DR    CTCCGCTGCGCGTTCCTCTAGTTCCCATGGAGCATTGCACCTCAGTCTTCTTTCAAATGTGTGATGCCGACAAGGACAAA  1746
SPARCl1˙IP    CTCCTCTGCGTGCCCCTCTGGTTCCTATGGAGCACTGCACTTCAGTTTTTTTCCACGAGTGTGATGCAGACAAGGACAAG  1752
SPARCl1˙LW    CTCCTCTGCGCGTTCCCTTGGTTCCCATGGAGCATTGCACCTCTGTCTTTTTCCAGAAGTGTGATGCTGACAAGGACAAA  1809
SPARCl1˙PN    CTCCTCTGCGTGTTCCATTGGTTCCCATGGAGCATTGCACTTCAGTCTTTTTCCAGGAATGCGACGCAGACAAGGACAAG  1737
SPARCl1˙PP    CTCCTCTGCGCGTTCCCCTGGTTCCCATGGAGCATTGCACCTCTGTCTTTTTCCAAAAGTGTGATGCTGACAAGAACAAA  1752
SPARCl1-AM    ................................................................................  1041
SPARCl1-C1˙CC CTCCACTGCGCGTCCCCCTAGTTCCAATGGAGCATTGCACCTCAGTCTTTTTCCAAAAGTGTGATGCTGACAAGGACAAA  1782
SPARCl1-C2˙CC TTCCATTGCGGGTTCCCCTAGTTCCAATGGAGCATTGCACCTCAGTCTTTTTCCAAAGGTGTGATGCTGACAAGGACAAA  1806
SPARCl1-C1˙SG CTCCACTGCGCGTTCCCCTAGTTCCAATGGAGCATTGCACCTCAGTCTTTTTCCAAAAGTGTGATGCTGACAAGGACAAA  1800
SPARCl1-C2˙SG CTCCACTGCGCGTTCCCCTAGTTCCAATGGAGCATTGCACCTCAGTCTTTTTCCAAAGATGTGATGCTGACAAGGACAAA  1800
SPARCl1-C1˙SA CTCCACTGCGCGTTCCCCTAGTTCCAATGGAGCATTGCACCTCAGTCTTTTTCCAAAAGTGTGATGCTGACAAGGACAAA  1800
SPARCl1-C2˙SA CTCCACTGCGCGTTCCCCTAGTTCCAATGGAGCATTGCACCTCAGTCTTTTTCCAAAGATGTGATGCTGACAAGGACAAA  1818
SPARCl1-C1˙SR CTCCACTGCGTGTTCCCCTAGTTCCAATGGAGCATTGTACCTCAGTCTTTTTCCAAAAGTGTGATGCTGACAAGGACAAA  1779
SPARCl1-C2˙SR CTCCACTGCGCGTTCCCCTAGTTCCAATGGACCATTGCACCTCAGTCTTTTTCCAAAGATGTGATGCTGACAAGGACAAA  1788

             1810      1815      1820      1825      1830      1835      1840      1845      1850      1855      1860      1865      1870      1875      1880
conservation
SPARCl1˙CI    CTAGTCTCCTTCAAAGAGTGGTGCAGCTGCTTTGGCATTAAGGAAGAGGATATGGATACCAATCTGCTGTTCTGA  1884
SPARCl1˙DR    CTGGTCTCCTTCAAAGAGTGGTGCAGCTGCTTTGGCATTAAGGAAGAGGATATGAATACCAATCTGCTGTTCTGA  1821
SPARCl1˙IP    CTACTCTCTTTCAGGGAGTGGTGCCAGTGTTTCGGCATCAAGGATGAGGACATGGACACCAAGCTTCTGTTCTAA  1827
SPARCl1˙LW    CTTGTCTCCTTCAAAGAGTGGTGCAGCTGCTTTGGCATTAAAGAAGAGGATATGGATTCTAATCTGCTGTTCTGA  1884
SPARCl1˙PN    CTGGTCTCCTTCAGGGAGTGGTGCCAATGTTTCGGCATCAAGGACGAGGACATGGATACCAAGCTGTTGTTCTAA  1812
SPARCl1˙PP    CTAGTCTCCTTCAAAGAGTGGTGCAGCTGCTTTGGCATTAAAGAAGAGGATATGGATTCTAATCTGCTGTTCTGA  1827
SPARCl1-AM    ...........................................................................  1041
SPARCl1-C1˙CC CTGGTCTCCTTCAAGGAGTGGTGCAGCTGCTTTGGCATTAAGGAAGATGATATGGATGCCAATCTGCTGTTCTGA  1857
SPARCl1-C2˙CC CTGGTCTCCTTCAAGGAGTGGTGCAGCTGCTTTGGCATTAAGGAAGAGAACATGGATACCAATCTGCTGTTTTGA  1881
SPARCl1-C1˙SG TTGGTCTCCTTCAAGGAGTGGTGCAGCTGCTTTGGCATTAAGGAAGAGGATATGGATGCCAATCTGCTGTTCTGA  1875
SPARCl1-C2˙SG CTGGTCTCCTTCAAGGAGTGGTGCAGCTGCTTTGGCATTAAGGAAGAGGACATGGATACCAATCTGCTGTTCTGA  1875
SPARCl1-C1˙SA TTGGTCTCCTTCAAGGAGTGGTGCAGCTGCTTTGGCATTAAGGAAGAGGATATGGATGCCAATCTGCTGTTCTGA  1875
SPARCl1-C2˙SA CTGGTCTCCTTCAAGGAGTGGTGCAGCTGCTTTGGCATTAAGGAAGAGGACATGGATACCAATCTGCTGTTCTGA  1893
SPARCl1-C1˙SR TTGGTCTCCTTCAAGGAGTGGTGCAGCTGCTTTGGCATTAAGGAAGAGGATATGGATGCCAATCTGCTGTTCTGA  1854
SPARCl1-C2˙SR CTGGTCTCCTTCAAGGAGTGGTGCAGCTGCTTTGGCATCAAGGAAGAGGACATGGATACCAATCTGCTGTTCTGA  1863
```

X non conserved
X ≥ 55% conserved
X ≥ 85% conserved
